# Cost-Effectiveness of Proton Beam Therapy for Intraocular Melanoma

**DOI:** 10.1371/journal.pone.0127814

**Published:** 2015-05-18

**Authors:** James P. Moriarty, Bijan J. Borah, Robert L. Foote, Jose S. Pulido, Nilay D. Shah

**Affiliations:** 1 Robert D. and Patricia E. Kern Center for the Science of Health Care Delivery, Mayo Clinic, Rochester, Minnesota, United States of America; 2 Division of Health Care Policy and Research, Mayo Clinic, Rochester, Minnesota, United States of America; 3 Department of Radiation Oncology, Mayo Clinic, Rochester, Minnesota, United States of America; 4 Department of Ophthalmology, Mayo Clinic, Rochester, Minnesota, United States of America; University of Alabama at Birmingham, UNITED STATES

## Abstract

**Purpose:**

Proton beam therapy is a commonly accepted treatment for intraocular melanomas, but the literature is lacking in descriptions of patient preferences of clinical outcomes and economic impact. In addition, no economic evaluations have been published regarding the incremental cost-effectiveness of proton beam therapy compared with enucleation or plaque brachytherapy, typical alternative treatments. We, therefore, conducted a cost-utility analysis of these three approaches for the treatment of intraocular melanomas.

**Materials and Methods:**

A Markov model was constructed. Model parameters were identified from the published literature and publicly available data sources. Cost-effectiveness of each treatment was calculated in 2011 US Dollars per quality-adjusted life-year. Incremental cost-effectiveness ratios were calculated assuming enucleation as reference. One-way sensitivity analyses were conducted on all model parameters. A decision threshold of $50,000/quality-adjusted life-year was used to determine cost-effectiveness.

**Results:**

Enucleation had the lowest costs and quality-adjusted life-years, and plaque brachytherapy had the highest costs and quality-adjusted life-years. Compared with enucleation, the base-case incremental cost-effectiveness ratios for plaque brachytherapy and proton beam therapy were $77,500/quality-adjusted life-year and $106,100/quality-adjusted life-year, respectively. Results were highly sensitive to multiple parameters. All three treatments were considered optimal, and even dominant, depending on the values used for sensitive parameters.

**Conclusion:**

Base-case analysis results suggest enucleation to be optimal. However, the optimal choice was not robust to sensitivity analyses and, depending on the assumption, both plaque brachytherapy and proton beam therapy could be considered cost-effective. Future clinical studies should focus on generating further evidence with the greatest parameter uncertainty to inform future cost-effectiveness analyses.

## Introduction

As the costs of health care have increased, the value, in terms of both costs and benefits, of the next generation, high-cost technologies such as robotic surgery[1,2], proton beam therapy[3–16], and biological drugs[17,18] has been debated. Proton beam therapy, in particular, has been a lightning rod in the debate regarding the incremental value of new technology in both the lay press and academic discussions.[19–21] The empirical evidence for proton beam therapy in the literature is limited in terms of descriptions of clinical outcomes and economic impact. In addition, much of the debate about proton beam therapy has been centered on its use for prostate cancer, with limited discussion of its potential benefits for numerous other indications. A systematic review assessing the role of proton beam therapy for various cancers suggested no difference in overall or cancer-specific survival, or even in adverse events, compared with conventional radiotherapy.[15]

One of these other cancers, intraocular melanoma, has been considered a “commonly accepted indication” for proton beam therapy.[7] A recent systematic review and meta-analysis by our group suggested that proton beam therapy for uveal melanoma is associated with better tumor control and fewer complications than plaque brachytherapy, although the quality of the evidence was low.[22] However, there are no published economic evaluations of the incremental cost-effectiveness of proton beam therapy compared with enucleation or plaque brachytherapy, which are standard alternative treatments for intraocular melanoma. The Collaborative Ocular Melanoma Study, which compared enucleation and plaque brachytherapy, indicated no difference between these treatments in terms of survival and tumor control.[23] In addition, few centers that have proton beam therapy have published their outcomes.[24–26] A randomized controlled trial comparing the outcomes of uveal melanoma after treatment with helium ions or plaque brachytherapy showed more local recurrences in the plaque brachytherapy group.[27]

Given the clinical benefit of proton beam therapy for intraocular melanoma, but lack of evidence of its economic impact, we conducted a cost-utility analysis of enucleation, plaque brachytherapy, and proton beam therapy for the treatment of intraocular melanomas.

## Methods

### Model Design

**Fig 1:**
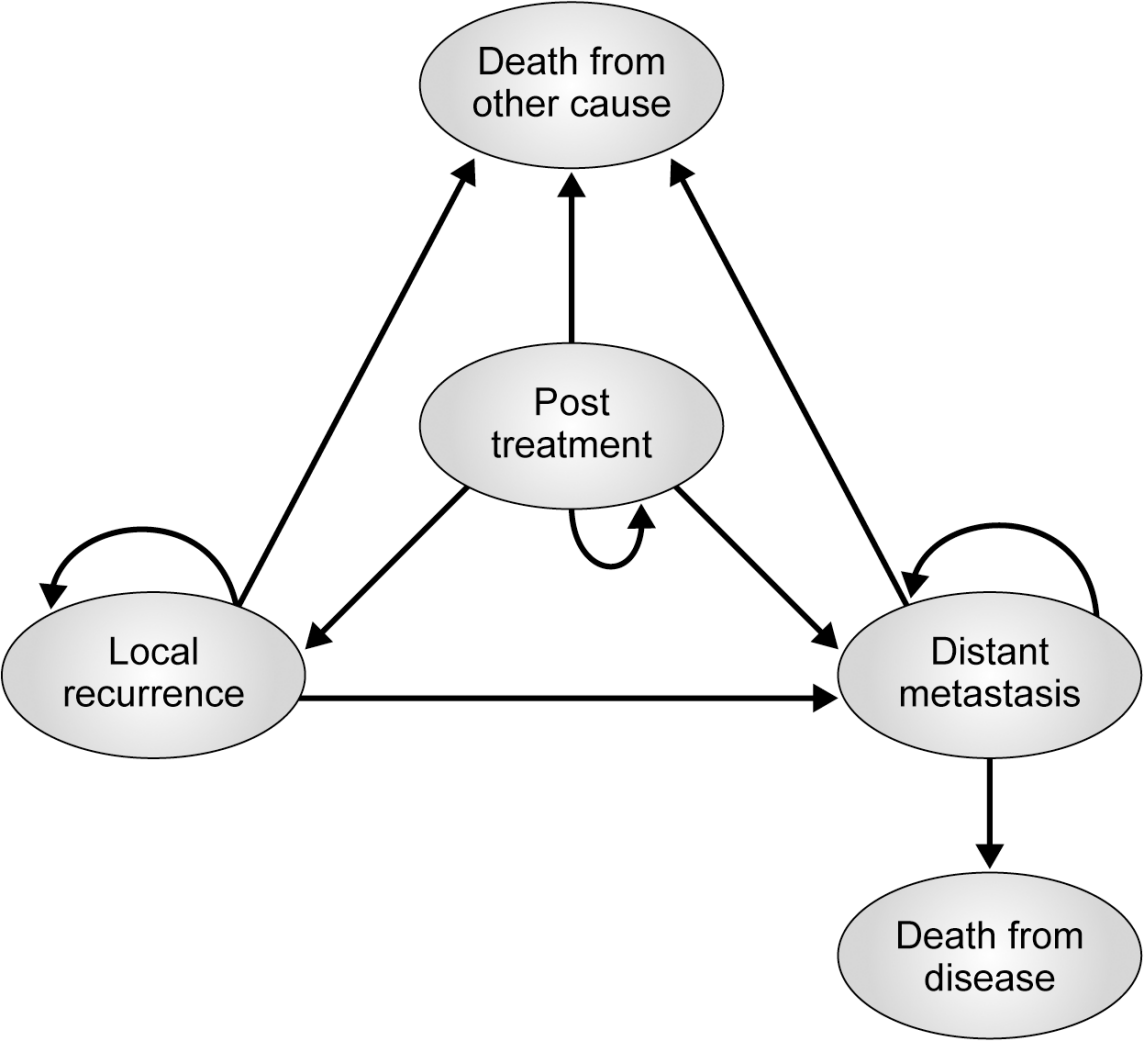
Markov Model schematic. Ovals signify the differing health states. Arrows indicate pathways that can occur. Arrows returning back to the same health state signify remaining in that health state. All individuals start in the “Post Treatment” health state.

A Markov model was constructed using TreeAge Pro 2012 (TreeAge Software). Three treatment options were compared: enucleation, plaque brachytherapy, and proton beam therapy. Five distinct health states were considered: post treatment, local recurrence, metastatic cancer, death due to disease, and death due to other causes. Pathways of the model are depicted in Fig 1.

The analyses consisted of a hypothetical cohort of 10,000 patients simulated through the model and repeated 1,000 times for each of the three treatment approaches. The time horizon of the model was five years, and each model cycle represented one year. Patients were assumed to be 59 years old at the start of the model, based on weighted mean age of the general intraocular melanoma population from previously published studies.[25,28–31] Patients who experienced a local recurrence were assumed to not be able to recover to the initial post treatment state. It was assumed that patients in the metastatic cancer state would stay in the state for one year and then die of disease.[32] To account for the uncertainty of timing for changing between health states, the half-cycle correction was used.[33] This assumes any change in health state happens halfway through the yearly cycle, thus averaging out the timing of these events.

### Ethics Statement

This study was deemed exempt from needing IRB approval.

### Probability Parameters

Treatment-specific probability parameters were obtained from a review of the published literature. The literature search focused on peer-reviewed published literature that could be used for health state probability parameters for the three treatment options. Abstracts of papers identified from the search were reviewed for relevance. Those deemed relevant had full text reviews. Additional key articles not found in the literature search were also reviewed for potential inclusion. Further details regarding the literature review and model inclusion are described in S1 File: Health State Probability Literature Search methods. Studies reporting results usable for the model are reported in Table 1. Studies reporting results based on tumor size or location in the eye were pooled together. Probabilities between health states were weighted by sample size. Care was taken to identify studies from research groups publishing results from overlapping populations; if two or more study populations overlapped, the study with the largest population was used to determine the parameter values for base-case analysis.

**Table 1:** Model Parameters: Health State Probabilities.

			Probabilities			
Treatment	Current Health State	Subsequent Health State	Base Case	Min	Max	References, Base Case
Proton beam therapy	Post treatment	Local recurrence	0.009	0.001	0.030[34]	[25,35,36]
		Metastasis	0.039	0.001[37]	0.083[38]	[24,25,30]
	Local recurrence	Metastasis	0.061	0.001[37]	0.202[39]	[24,25,30,39] Assumption†
Plaque brachytherapy	Post treatment	Local recurrence	0.021	0.001	0.030[34]	[25,34,38,40–42]
		Metastasis	0.029	0.001[37]	0.083[38]	[34,38,40,42,43]
	Local recurrence	Metastasis	0.045	0.001[37]	0.202[39]	[34,38,40,42,43] Assumption†
Enucleation	Post treatment	Local recurrence	0.002	0.001	0.030[34]	[44]
		Metastasis	0.047	0.001[37]	0.083[38]	[29,43]
	Local recurrence	Metastasis	0.074	0.001[37]	0.202[39]	[29,43] Assumption†

Abbreviations: Max, maximum; Min, minimum.

† Base-case transition probability values for transitioning from local recurrence to metastasis were assumed to have a relative risk ratio of 1.5 compared with the transition probability of transitioning from post treatment to metastasis.

Only one identified study contained usable data for probability parameters for patients with local recurrence and subsequent development of metastatic cancer. Although that study only involved plaque brachytherapy, we assumed this relative risk to be the same for patients undergoing enucleation or proton beam therapy. Therefore, the relative risk ratio of 1.5 reported by Jampol et al[41] was applied to the probability parameters of post treatment to metastasis. Probability of death due to other causes was based on age-specific mortality probabilities from the 2008 US Life Tables from the National Vital Statistics database.[45] The probability of remaining in the post treatment or local recurrence state was calculated as 1.0 minus the probabilities of the other potential pathways.

### Cost Parameters

Only direct medical costs were used as inputs for the model using the provider perspective. To date, few data have been published pertaining to treatment costs (direct and indirect), downstream costs of disease progression and acute and late toxicity, or costs associated with loss of an eye and loss of vision for intraocular melanoma. For this reason, publicly available databases and Medicare reimbursement rates of specific services were investigated as possible data sources (Table 2). To account for well-known discrepancies between billed charges and actual resource use, charges were converted to costs using cost-to-charge ratios. [46]

**Table 2:** Model Parameters: Cost and Utility Parameters.

Parameter				Value			
				Base Case	Min	Max	Reference
Cost Type, $							
	Treatment						
		Enucleation		$8,678	$6,075	$13,364	[47]
		Plaque brachytherapy					
			Total	$19,108	$13,376	$29,426	
			Hospital	$16,444	NA	NA	[48]
			Physician	$1,778	NA	NA	Medicare reimbursement (See S1 Table)
		Proton beam therapy		$12,438	$8,707	$19,155	Medicare reimbursement (See S1 Table)
	Local recurrence						
		Enucleation		$8,678	$6,075	$13,364	Assumption†
		Plaque brachytherapy		$8,678	$6,075	$13,364	[47]
		Proton beam therapy		$8,678	$6,075	$13,364	[47]
	End-of-life costs						
		Disease		$76,645	$53,652	$118,035	[49]
		Other causes		$42,787	$29,952	$65,894	[50]
Health State Utilities							
	Post treatment						
		Enucleation		0.71	0.66	0.76	[51]
		Plaque brachytherapy		0.71	0.66	0.76	[51]
		Proton beam therapy		0.706	0.656	0.756	[52]
	Local recurrence			0.52	0.47	0.57	[53]
	Metastasis			0.23	0.18	0.28	[53]

Abbreviations: Max, maximum; Min, minimum; NA, not applicable.

† Costs of local recurrence for enucleation could not be found in the literature. We assumed the cost of recurrence would be equivalent to costs of enucleation for initial treatment.

Inpatient treatment costs of plaque brachytherapy comprised both hospital costs and physician services. Hospital costs were obtained from the 2010 Nationwide Inpatient Sample (NIS) database. The NIS database is the largest all-payer inpatient care database in the United States, containing approximately one-fifth of all inpatient admissions from a stratified sample.[48] It is a part of the Healthcare Cost and Utilization Project (HCUP), which is sponsored by the Agency for Healthcare Research and Quality. Relevant inpatient episodes were identified in the NIS by the ICD-9 procedure code 14.27, as either primary or secondary procedure codes. Financial data in the NIS consists of billed charges for direct medical services provided. Mean costs were estimated from charges using cost-to-charge ratio files provided by HCUP.[54] Costs of physician services for plaque brachytherapy were valued using Medicare reimbursement rates (S1 Table). Proton beam therapy costs were valued using Medicare reimbursement rates based on CPT-4 billing codes (S1 Table). For both plaque brachytherapy and proton beam therapy the quantity billed of each CPT-4 code is the number of times each CPT code is charged to a single patient during their complete course of plaque brachytherapy or proton beam therapy. Simulated patients having plaque brachytherapy or proton beam therapy who had local recurrence were assumed to undergo enucleation for the recurrence. Patients with a local recurrence after enucleation were assumed to be treated with conventional radiotherapy. Radiotherapy costs could not be identified; consequently, costs for enucleation were used as a proxy. End-of-life costs of metastatic cancer were valued using a study reporting mean costs in the final six months of life for patients dying of metastatic cancer.[49] Cost parameters for a given health state were assumed to occur when a simulated patient entered the health state. Recurring cycles in a health state would not result in additional costs for that health state. All cost parameters were valued in 2011 US Dollars and were inflated to that year using the gross domestic product price deflator.[55,56] To reflect time preferences for resources, future costs produced by the model were discounted to present value using the standard rate of 3%.[57]

### Quality of Life

Quality of life for each health state was valued using utility measurements. This approach values the quality of life in different health states based on individual preferences.[58] Quality of life is reported using quality-adjusted life-years (QALYs). This unit of measurement treats one year of life in perfect health as a QALY of 1.0, whereas one year of life in lesser health states would be some fraction of one QALY. Utility values of each health state are depicted in Table 2. Few studies have reported quality-of-life results for intraocular melanoma patients in a manner that can be converted to QALYs. No studies were found reporting quality of life associated with local recurrence or metastasis of intraocular melanoma. As an alternative, local recurrence and metastasis values were taken from a study reporting on general melanoma patients.[53] As with cost outcomes, future QALYs were discounted at 3%.[57]

### Base-Case Analysis

Parameter values were held constant for base-case simulations. Primary outcomes included mean costs, mean QALYs, and incremental cost-effectiveness ratios (ICERs) using enucleation as reference. An incremental cost-effectiveness threshold of $50,000/QALY was used to classify a comparator as cost-effective relative to enucleation.[59] In essence, the threshold value is used to determine at what additional cost for improved outcomes is no longer a good value. Secondary outcomes included mean percentage of patients ending in each health state (including the death states) and mean percentages of patients ever having a local recurrence or metastasis. Furthermore, average time in each health state was determined.

### Sensitivity Analysis

To assess uncertainty in model parameters for cost-effectiveness, one-way sensitivity analysis was performed on all parameters. This approach varies the value of one parameter while all other parameters are held constant at their base-case value. Low and high values for probabilities were based on unweighted low and high values of studies available for the base-case analysis (Table 1). Parameter information from studies deemed to overlap, and which were excluded from base-case, were eligible for sensitivity analysis. Because tumor size and location often influence treatment choice for intraocular melanoma and because tumor characteristics can affect health state probability values, we decided that sensitivity analysis of probability parameters would not be based on treatment-specific values. Consequently, the probability parameter low and high values were identical for all three treatment approaches. This was thought to be the most conservative approach in identifying parameters that influence model results. Additionally, the lowest value found for the annual probability of local recurrence was 0.006, which was close to the base-case value for proton beam therapy. To be conservative, we used a value of 0.001 for the low value of local recurrence probability.

Low and high values for sensitivity analysis of cost parameters were determined by using reported ranges of Medicare spending per beneficiary; base-case cost values were weighted by low (0.70) and high (1.54) values of Medicare spending per beneficiary. Whereas values for costs of local recurrence were assumed to be equivalent for all three treatments, sensitivity analysis of this parameter for enucleation was done separately from that of plaque brachytherapy and proton beam therapy.

One-way sensitivity analyses were performed using ICER thresholds of $50,000/QALY. The range of ICERs in sensitivity analyses were determined to describe the variability of model results from sensitivity analyses.

## Results

### Base-Case Analysis

Base-case analysis showed that enucleation had the lowest mean costs and lowest mean QALYs, at $22,772 and 2.918, respectively. Plaque brachytherapy had the highest mean costs at $28,662 but also the highest mean QALYs at 2.994, resulting in an ICER of $77,500/QALY. Mean proton beam therapy costs were $24,894 and mean QALYs were 2.938. The ICER of proton beam therapy compared with enucleation was $106,100/QALY. Neither plaque brachytherapy nor proton beam therapy had an ICER less than the cost-effectiveness threshold of $50,000/QALY. Simulation results for health state information is given in Table 3. For all three treatment approaches, similar percentages of individuals, ranging from 73.7% to 74.5%, ended in the post treatment state. Plaque brachytherapy had the largest percentage of individuals ending the simulation in the local recurrence state (8.3%) and enucleation had the smallest (0.7%). Conversely, enucleation had the greatest percentage of individuals ending in the metastatic state (3.8%) and dying of disease (17.3%). Plaque brachytherapy had the fewest individuals ending in the metastatic state (2.6%) and dying of disease (11.1%). Similar results were seen with average time spent in each health state and total time in the model (Table 4). On average, patients spent approximately 4.3 years in the post treatment state and lived 4.6 years of the 5-year simulation.

**Table 3:** Final Health States, Local Recurrence, and Metastasis.

Variable		Therapy^†^		
		Enucleation	Plaque Brachytherapy	Proton Beam Therapy
Ending state				
	Post treatment	74.1 (72.4–74.9)	73.7 (72.8–74.5)	74.5 (73.6–75.3)
	Local recurrence	0.7 (0.6–0.9)	8.3 (7.8–8.8)	3.5 (3.1–3.8)
	Metastasis	3.8 (3.4–4.1)	2.6 (2.3–2.9)	3.3 (3.0–3.6)
	Death of disease	17.3 (16.6–18.1)	11.1 (10.5–11.7)	14.6 (13.9–15.4)
	Death of other causes	4.1 (3.7–4.5)	4.3 (3.9–4.7)	4.2 (3.8–4.6)
Ever local recurrence		0.9 (0.7–1.1)	9.3 (8.8–9.9)	4.0 (3.7–4.4)
Ever metastasis		21.1 (20.3–21.9)	13.7 (13.1–14.4)	17.9 (17.2–18.6)

^†^ Values are mean percentage of patients (95% CI).

**Table 4:** Time in Each Health State and Total Time in Model for a Simulated Patient

Variable	Therapy^†^		
	Enucleation	Plaque Brachytherapy	Proton Beam Therapy
Post treatment	4.326 (4.300–4.352)	4.315 (4.289–4.341)	4.337 (4.312–4.363)
Local recurrence	0.021 (0.016–0.026)	0.229 (0.213–0.244)	0.096 (0.087–0.107)
Metastasis	0.192 (0.184–0.200)	0.124 (0.118–0.130)	0.163 (0.156–0.170)
Total time	4.539 (4.518–4.559)	4.668 (4.649–4.685)	4.596 (4.576–4.615)

^†^ Values are mean time (95% CI) in years.

### One-Way Sensitivity Analyses

The ICERs for one-way sensitivity analyses are given in Table 5. Eight parameters had no effect on overall results of the model: probability of metastasis from local recurrence for enucleation, plaque brachytherapy, and proton beam therapy; end-of-life costs for other causes; cost of local recurrence for enucleation and proton beam therapy or plaque brachytherapy; local recurrence utility; and metastasis utility. Model results were sensitive to the remaining 13 parameters. These included probability of local recurrence for all three treatment approaches, probability of metastasis from post treatment for all three treatment approaches, end-of-life costs for disease, treatment costs for all three treatment approaches, and post treatment utility for all three treatment approaches.

**Table 5:** One-Way Sensitivity Analysis.

Parameter			ICER, $^†^			
			Low Parameter Value		High Parameter Value	
			Plaque Brachytherapy	Proton Beam Therapy	Plaque Brachytherapy	Proton Beam Therapy
LR probability						
	Enucleation^‡^		80,162	119,491	35,489	11,881
	Plaque brachytherapy^‡^		43,414	105,306	105,898	105,306
	Proton beam therapy^‡^		77,434	49,198	77,434	Dominated
Met probability						
	Enucleation^‡^		Dominated	Dominated	Dominates	Dominates
	Plaque brachytherapy^‡^		Dominates	105,900	Dominated	105,900
	Proton beam therapy^‡^		77,434	Dominates	77,434	Dominated
Met probability from LR						
	Enucleation		78,158	111,939	74,385	95,975
	Plaque brachytherapy		68,284	105,306	112,597	105,306
	Proton beam therapy		77,434	84,168	77,434	172,785
End-of-life costs						
	Other causes		77,197	104,900	77,894	106,036
	Disease^‡^		94,868	134,014	46,066	53,628
Treatment cost						
	Enucleation^‡^		111,684	234,682	15,776	Dominates
	Plaque brachytherapy^‡^		13,558	105,900	224,855	105,900
	Proton beam therapy^‡^		77,434	Dominates	77,434	441,750
Cost of LR						
	Enucleation		66,618	106,397	66,618	103,341
	Plaque brachytherapy		74,408	100,385	82,882	114,165
	Proton beam therapy		74,408	100,385	82,882	114,165
Utility						
	Post treatment					
		Enucleation^‡^	21,169	9,543	Dominated	Dominated
		Plaque brachytherapy^‡^	Dominated	105,306	21,169	105,306
		Proton beam therapy^‡^	77,434	Dominated	77,434	9,522
	LR		87,836	126,869	68,430	90,008
	Met		74,494	98,636	79,527	112,943

Abbreviation: ICER, incremental cost-effectiveness ratio; LR, Local recurrence; Met, Metastasis.

^†^ Enucleation is base case.

^‡^ Parameter in which model results are sensitive to change from low to high values.

In total, the ICERs for plaque brachytherapy and proton beam therapy compared with enucleation varied considerably resulting in the most cost-effective treatment to change depending on the parameter value (Table 5). For low values of model parameters, the ICERs for plaque brachytherapy and proton beam therapy ranged from $21,169/QALY to $111,684/QALY and $9,543/QALY to $234,682/QALY, respectively. There was even greater variability in ICERs of high values for model parameters; plaque brachytherapy ICERs ranged from $15,776/QALY to $224,855/QALY and proton beam ICERs ranged from $9,522/QALY to $441,750/QALY. Of note, both plaque brachytherapy and proton beam therapy had instances of being dominant (less costly and better outcomes) compared with enucleation, or dominated (more costly and worse outcomes) by enucleation when evaluating both low and high values of model parameters. Across low and high values for model parameters, plaque brachytherapy dominated enucleation in two instances and was dominated by enucleation in four instances. Similarly, proton beam therapy dominated enucleation in four instances and was dominated by enucleation in five instances.

## Discussion

The results of this base-case analysis suggest that plaque brachytherapy and proton beam therapy are not cost-effective alternatives to enucleation as their respective ICERs are beyond the threshold value of $50,000/QALY. However, mean cost differences were minimal among the three treatment approaches, and mean QALY differences were extremely small. These extremely small differences in QALYs was the driving force in the ICERs being greater than $50,000/QALY for both plaque brachytherapy and proton beam therapy.

Sensitivity analyses revealed model results to be sensitive to a sizable proportion of model parameters. Furthermore, comparing plaque brachytherapy or proton beam therapy with enucleation, the range of ICERs varied considerably. Consequently, sensitivity analyses show each of the three treatment options to be cost-effective, and even dominant, depending on specific values of key model parameters. This high degree of uncertainty in which treatment is most cost-effective is our key finding. We would recommend that future studies of intraocular melanoma treatment include detailed quality-of-life end points, as well as focus on parameters with the greatest uncertainty in this analysis, to provide more robust data to be included in future cost-effectiveness research. This could include utility data regarding the cosmetic value of having an eye versus enucleation, and the value of having normal, partial, or no vision in an eye. Both provider- and patient-reported outcomes would be valuable.

Much of the discussion on the value of proton beam therapy has focused around its use for early-stage prostate cancer. In our model, we find that proton beam therapy can be cost-effective, dominant, or not cost-effective depending on the parameter estimates. This further highlights the lack of empirical comparative evidence for proton beam therapy that has been highlighted in previous systematic reviews.[15,60] More recently, Medicare has decreased payments for proton beam therapy.[61] As shown in the sensitivity analysis, these changes could make proton beam therapy the preferred treatment because it would dominate both enucleation and plaque brachytherapy. Similar issues exist with the use of plaque brachytherapy. The cost-effectiveness of plaque brachytherapy varied compared with both enucleation and proton beam therapy in the sensitivity analysis.

End-of-life costs far outweigh the cost of any form of treatment in our analysis. Therefore, it is important to measure not only the near-term efficacy of these treatments, but also whether end-of-life costs vary depending on the treatment used. Availability of better estimates for this aspect of care would significantly enhance findings from this model.

### Limitations

This study has several limitations. First, treatment choice is often influenced by factors that would also influence health state probability parameters. Although this would certainly affect the base-case results, we designed our sensitivity analyses to not be treatment specific by including minimum and maximum values of health state probability parameters, which would help mitigate this issue by giving a wider range of possible values. Next, all isotope types of plaque brachytherapy were considered in data collection. Different isotopes may result in differing health state probabilities. We also did not conduct a full systematic review of the literature to obtain model parameters. In addition, studies identified as overlapping were not verified to be the case by each publishing study group. Next, relative risk ratios for patients with local recurrence followed by distant metastasis came from a study of patients treated with plaque brachytherapy. Our base-case results assume this relative risk ratio to be identical for patients treated with enucleation and proton beam therapy, which may not be the case. However, the upper limit on sensitivity analyses for each treatment was much higher, and none of these parameters proved to influence results.

The cost analysis also has some limitations. Our study used disease-reported costs for the last 6 months of life rather than the entire final year, which would then underestimate costs in the final year. Unfortunately, few reports have shown the exact costs for the final year of life. We believed that underestimating this parameter would be more palatable than doubling the costs and risking their overestimation. We also had to use a proxy for costs for treatment of a local recurrence after initial enucleation. However, this parameter was not sensitive to the model results. Finally, very little data are available for analysis of the indirect medical costs associated with each form of treatment, the costs of treating acute and late complications associated with each form of treatment, the utility or value of retaining an eye with or without useful vision, including functional and cosmetic outcomes, provider- or patient-reported quality of life and functional outcomes, and the costs associated with radiation exposure of staff handling radioactive plaques. However, even given this limitation the results of this analysis can still aide decision makers by logically synthesizing the best available information. Without which, decisions regarding the appropriate use of limited resources must still be made.[62] These missing data should be collected prospectively to provide more robust cost-effectiveness analyses in the future to improve the available information for decision making.

## Conclusion

With data currently available, our base-case analysis suggests that the cost-effectiveness of both plaque brachytherapy and proton beam therapy is higher than the traditional threshold of $50,000/QALY. However, the results are sensitive to several parameters and could change significantly by changes in payment rates or better evidence on the comparative efficacy of these treatments, as well as the quality of life experienced by patients receiving each therapy. Further research on the sensitive parameters would be warranted.

## Supporting Information

S1 File Health State Probability Literature Search methods. (DOCX)

S1 Table CPT-4 Codes Used for Medicare Reimbursement Rates for Cost Parameters (DOCX)
