# Supplementary material for: Cost-Effectiveness of Proton Beam Therapy for Intraocular Melanoma
Source: PLoS One. 2015 May 18;10(5):e0127814. doi: 10.1371/journal.pone.0127814 (PMC4436277; doi:10.1371/journal.pone.0127814)
Supplement: S1 File — (DOCX) [file pone.0127814.s001.docx]

**S1 File.** Health State Probability Literature Search Methods

Four study groups were used as the basis of the literature search on health state probabilities. The study groups were denoted by a key author or authors or by the name of the study team. Search strategy and the number of articles produced are shown below in Supplementary Table 1.

**Table 1 in S1 File**. Search Criteria and References for Each Study Group

| **Study Group** | **Search Criteria** | **Articles Found** |
| --- | --- | --- |
| Shields | Shields CL and Shields JA as authors, cross-referenced with keywords ocular melanoma or uveal melanoma or choroidal melanoma | 170 (1-170) |
| Damato | Damato BE as author | 95 (171-265) |
| Gragoudas | Gragoudas ES as author, cross-referenced with keywords ocular melanoma or uveal melanoma or choroidal melanoma | 80 (266-345) |
| COMS | Collaborative Ocular Melanoma Study or COMS in article title | 81 (346-426) |

The literature search was performed in Ovid on August 22, 2012. Relevant articles with full text review are shown in Supplementary Table 2. The reason for exclusion is provided. Articles having potentially usable data for inclusion are also detailed. Additional key articles not found in the literature search were also eligible for potential study inclusion (427-433)

**Table 2 in S1 File.** Articles with Full Text Review and Inclusion/Exclusion Status

| **Study Group** | **Reason for Exclusion or Inclusion Details** |
| --- | --- |
| Shields (74) | No usable outcomes reported |
| Shields (60) | Eligible for use in study |
| Shields (73) | Data not reported in a usable manner |
| Shields (35) | No usable outcomes reported |
| Shields (59) | Data not reported in a usable manner |
| Shields (58) | Eligible for use in study |
| Shields (83) | Eligible for use in study |
| Shields (93) | No usable outcomes reported |
| Shields (159) | No usable outcomes reported |
| Shields (24) | Eligible for use in study |
| Shields (25) | No usable outcomes reported |
| Shields (26) | Eligible for use in study |
| Shields (63) | Data not reported in a usable manner |
| Shields (10) | Eligible for use in study |
| Shields (11) | Data not reported in a usable manner |
| Shields (106) | Data not reported in a usable manner |
| Shields (105) | No usable outcomes reported |
| Damato (220) | Data not reported in a usable manner |
| Damato (196) | Data not reported in a usable manner |
| Damato (197) | No usable outcomes reported |
| Damato (202) | Data not reported in a usable manner |
| Damato (201) | Data not reported in a usable manner |
| Damato (208) | Eligible for use in study |

**Table 2 in S1 File** (continued)

| **Study Group** | **Reason for Exclusion or Inclusion Details** |
| --- | --- |
| Damato (209) | Data not reported in a usable manner |
| Gragoudas (311) | Eligible for use in study |
| Gragoudas (339) | No usable outcomes reported |
| Gragoudas (303) | Eligible for use in study |
| Gragoudas (295) | No usable outcomes reported |
| Gragoudas (307) | No usable outcomes reported |
| Gragoudas (306) | Eligible for use in study |
| Gragoudas (285) | Eligible for use in study |
| Gragoudas (286) | No usable outcomes reported |
| Gragoudas (278) | Eligible for use in study |
| Gragoudas (318) | No usable outcomes reported |
| Gragoudas (283) | Eligible for use in study |
| Gragoudas (280) | No usable outcomes reported |
| Gragoudas (269) | Eligible for use in study |
| Gragoudas (314) | No usable outcomes reported |
| Gragoudas (332) | No usable outcomes reported |
| COMS (374) | Data not reported in a usable manner |
| COMS (363) | No usable outcomes reported |
| COMS (362) | Eligible for use in study |
| COMS (408) | Data not reported in a usable manner |
| COMS (382) | Data not reported in a usable manner |
| COMS (395) | Eligible for use in study |
| COMS (397) | Eligible for use in study |
| COMS (379) | Eligible for use in study |

**Table 2 in S1 File** (continued)

| **Study Group** | **Reason for Exclusion or Inclusion Details** |
| --- | --- |
| COMS (406) | No usable outcomes reported |
| COMS (350) | Eligible for use in study |
| COMS (351) | Data not reported in a usable manner |
| COMS (352) | No usable outcomes reported |
| COMS (348) | No usable outcomes reported |
| Added (430) | Eligible for use in study |
| Added (432) | Eligible for use in study |
| Added (429) | Eligible for use in study |
| Added (428) | Eligible for use in study |
| Added (433) | Eligible for use in study |
| Added (431) | Eligible for use in study |
| Added (427) | Eligible for use in study |

**References**

1. Aoyama T, Mastrangelo MJ, Berd D, Nathan FE, Shields CL, Shields JA, et al. Protracted survival after resection of metastatic uveal melanoma. Cancer. 2000;89(7):1561-8. Epub 2000/10/03. PubMed PMID: 11013372.

2. Arevalo JF, Shields CL, Shields JA. Giant nodular posterior scleritis simulating choroidal melanoma and birdshot retinochoroidopathy. Ophthalmic Surg Lasers Imaging. 2003;34(5):403-5. Epub 2003/09/26. PubMed PMID: 14509467.

3. Bianciotto C, Demirci H, Shields CL, Eagle RC, Jr., Shields JA. Metastatic tumors to the eyelid: report of 20 cases and review of the literature. Arch Ophthalmol. 2009;127(8):999-1005. Epub 2009/08/12. doi: 10.1001/archophthalmol.2009.120. PubMed PMID: 19667336.

4. Bianciotto C, Shields CL, Pirondini C, Mashayekhi A, Furuta M, Shields JA. Proliferative radiation retinopathy after plaque radiotherapy for uveal melanoma. Ophthalmology. 2010;117(5):1005-12. Epub 2010/01/19. doi: 10.1016/j.ophtha.2009.10.015. PubMed PMID: 20079924.

5. Bianciotto C, Shields CL, Pirondini C, Mashayekhi A, Furuta M, Shields JA. Vitreous hemorrhage after plaque radiotherapy for uveal melanoma. Retina. 2012;32(6):1156-64. Epub 2012/03/01. doi: 10.1097/IAE.0b013e3182340cc1. PubMed PMID: 22366905.

6. Brady LW, Shields JA, Shields CL, Glennon PT, Hernandez JC. Organ preservation: choroidal melanoma treated by brachytherapy techniques. Front Radiat Ther Oncol. 1993;27:1-19. Epub 1993/01/01. PubMed PMID: 8504938.

7. Connolly BP, Regillo CD, Eagle RC, Jr., Shields CL, Shields JA, Moran H. The histopathologic effects of transpupillary thermotherapy in human eyes. Ophthalmology. 2003;110(2):415-20. Epub 2003/02/13. doi: 10.1016/s0161-6420(02)01561-0. PubMed PMID: 12578790.

8. De Potter P, Flanders AE, Shields JA, Shields CL, Gonzales CF, Rao VM. The role of fat-suppression technique and gadopentetate dimeglumine in magnetic resonance imaging evaluation of intraocular tumors and simulating lesions. Arch Ophthalmol. 1994;112(3):340-8. Epub 1994/03/01. PubMed PMID: 8129659.

9. De Potter P, Shields CL, Shields JA. New treatment modalities for uveal melanoma. Curr Opin Ophthalmol. 1996;7(3):27-32. Epub 1996/05/07. PubMed PMID: 10163456.

10. De Potter P, Shields CL, Shields JA, Cater JR, Brady LW. Plaque radiotherapy for juxtapapillary choroidal melanoma. Visual acuity and survival outcome. Arch Ophthalmol. 1996;114(11):1357-65. Epub 1996/11/01. PubMed PMID: 8906026.

11. De Potter P, Shields CL, Shields JA, Cater JR, Tardio DJ. Impact of enucleation versus plaque radiotherapy in the management of juxtapapillary choroidal melanoma on patient survival. Br J Ophthalmol. 1994;78(2):109-14. Epub 1994/02/01. PubMed PMID: 8123617; PubMed Central PMCID: PMC504711.

12. De Potter P, Shields CL, Shields JA, Singh AD. Use of the hydroxyapatite ocular implant in the pediatric population. Arch Ophthalmol. 1994;112(2):208-12. Epub 1994/02/01. PubMed PMID: 8311774.

13. De Potter P, Shields JA, Shields CL, Santos R. Modified enucleation via lateral orbitotomy for choroidal melanoma with orbital extension: a report of two cases. Ophthal Plast Reconstr Surg. 1992;8(2):109-13. Epub 1992/01/01. PubMed PMID: 1520651.

14. De Potter P, Shields JA, Shields CL, Yannuzzi LA, Fisher YE, Rao VM. Unusual MRI findings in metastatic carcinoma to the choroid and optic nerve: a case report. Int Ophthalmol. 1992;16(1):39-44. Epub 1992/01/01. PubMed PMID: 1537648.

15. Demirci H, Shields CL, Honavar SG, Shields JA, Bardenstein DS. Long-term follow-up of giant nodular posterior scleritis simulating choroidal melanoma. Arch Ophthalmol. 2000;118(9):1290-2. Epub 2000/09/12. PubMed PMID: 10980778.

16. Demirci H, Shields CL, Shields JA, Eagle RC, Jr., Honavar S. Ring melanoma of the anterior chamber angle: a report of fourteen cases. Am J Ophthalmol. 2001;132(3):336-42. Epub 2001/09/01. PubMed PMID: 11530045.

17. Demirci H, Shields CL, Shields JA, Eagle RC, Jr., Honavar SG. Bilateral breast metastases from choroidal melanoma. Am J Ophthalmol. 2001;131(4):521-3. Epub 2001/04/09. PubMed PMID: 11292427.

18. Demirci H, Shields CL, Shields JA, Honavar SG, Eagle RC, Jr. Ring melanoma of the ciliary body: report on twenty-three patients. Retina. 2002;22(6):698-706; quiz 852-3. Epub 2002/12/12. PubMed PMID: 12476094.

19. Eagle RC, Jr., Ehya H, Shields JA, Shields CL. Choroidal metastasis as the initial manifestation of a pigmented neuroendocrine tumor. Arch Ophthalmol. 2000;118(6):841-5. Epub 2000/06/24. PubMed PMID: 10865324.

20. Furuta M, Shields CL, Uysal Y, Shields JA. Bilateral primary choroidal melanoma treated with bilateral plaque radiotherapy: a report of three cases. Eur J Ophthalmol. 2006;16(6):879-82. Epub 2006/12/28. PubMed PMID: 17191200.

21. Ghassemi F, Shields CL, Palamar M, Eagle RC, Jr., Shields JA. Black tears (melanodacryorrhea) from uveal melanoma. Arch Ophthalmol. 2008;126(8):1166-8. Epub 2008/08/13. doi: 10.1001/archopht.126.8.1166. PubMed PMID: 18695121.

22. Gunduz K, Shields CL, Shields JA. Varix of the vortex vein ampulla simulating choroidal melanoma: report of four cases. Retina. 1998;18(4):343-7. Epub 1998/09/08. PubMed PMID: 9730178.

23. Gunduz K, Shields CL, Shields JA, Cater J, Brady L. Plaque radiotherapy for management of ciliary body and choroidal melanoma with extraocular extension. Am J Ophthalmol. 2000;130(1):97-102. Epub 2000/09/27. PubMed PMID: 11004266.

24. Gunduz K, Shields CL, Shields JA, Cater J, Freire JE, Brady LW. Radiation complications and tumor control after plaque radiotherapy of choroidal melanoma with macular involvement. Am J Ophthalmol. 1999;127(5):579-89. Epub 1999/05/20. PubMed PMID: 10334352.

25. Gunduz K, Shields CL, Shields JA, Cater J, Freire JE, Brady LW. Radiation retinopathy following plaque radiotherapy for posterior uveal melanoma. Arch Ophthalmol. 1999;117(5):609-14. Epub 1999/05/18. PubMed PMID: 10326957.

26. Gunduz K, Shields CL, Shields JA, Cater J, Freire JE, Brady LW. Plaque radiotherapy of uveal melanoma with predominant ciliary body involvement. Arch Ophthalmol. 1999;117(2):170-7. Epub 1999/02/26. PubMed PMID: 10037560.

27. Gunduz K, Shields CL, Shields JA, Eagle RC, Jr., Singh AD. Iris mammillations as the only sign of ocular melanocytosis in a child with choroidal melanoma. Arch Ophthalmol. 2000;118(5):716-7. Epub 2000/05/18. PubMed PMID: 10815167.

28. Gunduz K, Shields CL, Shields JA, Schubert H. Presumed choroidal granuloma with vitreous hemorrhage resembling choroidal melanoma. Ophthalmic Surg Lasers. 1998;29(5):422-5. Epub 1998/05/26. PubMed PMID: 9599367.

29. Gunduz K, Shields JA, Shields CL, Eagle RC, Jr. Choroidal melanoma in a 14-year-old patient with ocular melanocytosis. Arch Ophthalmol. 1998;116(8):1112-4. Epub 1998/08/26. PubMed PMID: 9715696.

30. Gunduz K, Shields JA, Shields CL, Sato T, Mastrangelo MJ. Surgical removal of solitary hepatic metastasis from choroidal melanoma. Am J Ophthalmol. 1998;125(3):407-9. Epub 1998/03/25. PubMed PMID: 9512169.

31. Gunduz K, Shields JA, Shields CL, Zhao DY. Choroidal melanoma in a patient with retinitis pigmentosa and Usher's syndrome. Br J Ophthalmol. 1998;82(7):847-8. Epub 1999/01/30. PubMed PMID: 9924392; PubMed Central PMCID: PMC1722695.

32. Honavar SG, Shields CL, Demirci H, Shields JA. Sclerochoroidal calcification: clinical manifestations and systemic associations. Arch Ophthalmol. 2001;119(6):833-40. Epub 2001/06/19. PubMed PMID: 11405834.

33. Honavar SG, Shields CL, Singh AD, Demirci H, Rutledge BK, Shields JA, et al. Two discrete choroidal melanomas in an eye with ocular melanocytosis. Surv Ophthalmol. 2002;47(1):36-41. Epub 2002/01/22. PubMed PMID: 11801268.

34. Honavar SG, Singh AD, Shields CL, Shields JA, Eagle RC, Jr. Iris melanoma in a patient with neurofibromatosis. Surv Ophthalmol. 2000;45(3):231-6. Epub 2000/11/30. PubMed PMID: 11094247.

35. Horgan N, Shields CL, Mashayekhi A, Salazar PF, Materin MA, O'Regan M, et al. Periocular triamcinolone for prevention of macular edema after plaque radiotherapy of uveal melanoma: a randomized controlled trial. Ophthalmology. 2009;116(7):1383-90. Epub 2009/06/02. doi: 10.1016/j.ophtha.2009.01.051. PubMed PMID: 19481812.

36. Horgan N, Shields CL, Mashayekhi A, Shields JA. Classification and treatment of radiation maculopathy. Curr Opin Ophthalmol. 2010;21(3):233-8. Epub 2010/04/16. doi: 10.1097/ICU.0b013e3283386687. PubMed PMID: 20393294.

37. Horgan N, Shields CL, Mashayekhi A, Teixeira LF, Materin MA, O'Regan M, et al. Periocular triamcinolone for prevention of macular edema after iodine 125 plaque radiotherapy of uveal melanoma. Retina. 2008;28(7):987-95. Epub 2008/08/14. doi: 10.1097/IAE.0b013e31816b3192. PubMed PMID: 18698302.

38. Horgan N, Shields CL, Mashayekhi A, Teixeira LF, Materin MA, Shields JA. Early macular morphological changes following plaque radiotherapy for uveal melanoma. Retina. 2008;28(2):263-73. Epub 2008/02/28. doi: 10.1097/IAE.0b013e31814b1b75. PubMed PMID: 18301032.

39. Horgan N, Shields CL, Swanson L, Teixeira LF, Eagle RC, Jr., Ganguly A, et al. Altered chromosome expression of uveal melanoma in the setting of melanocytosis. Acta Ophthalmol. 2009;87(5):578-80. Epub 2008/06/13. doi: 10.1111/j.1755-3768.2008.01235.x. PubMed PMID: 18547285.

40. Hykin PG, Shields CL, Shields JA, Arevalo JF. The efficacy of focal laser therapy in radiation-induced macular edema. Ophthalmology. 1998;105(8):1425-9. Epub 1998/08/26. doi: 10.1016/s0161-6420(98)98023-x. PubMed PMID: 9709753.

41. Kheterpal S, Shields JA, Shields CL, De Potter P, Ehya H, Eng KY. Choroidal melanoma in an African-American albino. Am J Ophthalmol. 1996;122(6):901-3. Epub 1996/12/01. PubMed PMID: 8956654.

42. Lally DR, Shields JF, Shields CL, Marr BP, Shields JA. Pigmented free-floating vitreous cyst in a child. J Pediatr Ophthalmol Strabismus. 2008;45(1):47-8. Epub 2008/02/22. PubMed PMID: 18286964.

43. Li HK, Shields CL, Mashayekhi A, Randolph JD, Bailey T, Burnbaum J, et al. Giant choroidal nevus clinical features and natural course in 322 cases. Ophthalmology. 2010;117(2):324-33. Epub 2009/12/09. doi: 10.1016/j.ophtha.2009.07.006. PubMed PMID: 19969359.

44. Lois N, Shields CL, Shields JA, De Potter P, Ramsey MS. Trifocal uveal melanoma. Am J Ophthalmol. 1997;124(6):848-50. Epub 1997/12/24. PubMed PMID: 9402837.

45. Lois N, Shields CL, Shields JA, Eagle RC, Jr., De Potter P. Cavitary melanoma of the ciliary body. A study of eight cases. Ophthalmology. 1998;105(6):1091-8. Epub 1998/06/17. doi: 10.1016/s0161-6420(98)96013-4. PubMed PMID: 9627662.

46. Manquez ME, Shields CL, Demirci H, Shields JA, Beer P, Peters G, 3rd. Choroidal melanoma in a teenager with Klippel-Trenaunay-Weber syndrome. J Pediatr Ophthalmol Strabismus. 2006;43(4):197-8. Epub 2006/08/19. PubMed PMID: 16915894.

47. Marr BP, Shields JA, Shields CL, Materin MA, Tuncer S. Uveal prolapse following cataract extraction simulating melanoma. Ophthalmic Surg Lasers Imaging. 2008;39(3):250-1. Epub 2008/06/18. PubMed PMID: 18556954.

48. Mashayekhi A, Shields C, Shields JA. Disappearance of retinal myelinated nerve fibers after plaque radiotherapy for choroidal melanoma. Retina. 2003;23(4):572-3. Epub 2003/09/16. PubMed PMID: 12972781.

49. Mashayekhi A, Shields CL, Lee SC, Marr BP, Shields JA. Retinal break and rhegmatogenous retinal detachment after transpupillary thermotherapy as primary or adjunct treatment of choroidal melanoma. Retina. 2008;28(2):274-81. Epub 2008/02/28. doi: 10.1097/IAE.0b013e318145abe8. PubMed PMID: 18301033.

50. Mashayekhi A, Tuncer S, Shields CL, Shields JA. Tumor-related lipid exudation after plaque radiotherapy of choroidal melanoma: the role of Bruch's membrane rupture. Ophthalmology. 2010;117(5):1013-23. Epub 2010/01/26. doi: 10.1016/j.ophtha.2009.10.018. PubMed PMID: 20097428.

51. Milman T, Hu DN, McCormick SA, Eagle RC, Jr., Crawford JB, Chin K, et al. Expression of neurotrophin receptors by retinoinvasive uveal melanoma. Melanoma Res. 2012;22(2):164-8. Epub 2012/02/22. doi: 10.1097/CMR.0b013e32835175ec. PubMed PMID: 22343487.

52. Ohta M, Berd D, Shimizu M, Nagai H, Cotticelli MG, Mastrangelo M, et al. Deletion mapping of chromosome region 9p21-p22 surrounding the CDKN2 locus in melanoma. Int J Cancer. 1996;65(6):762-7. Epub 1996/03/15. doi: 10.1002/(sici)1097-0215(19960315)65:6<762::aid-ijc9>3.0.co;2-x. PubMed PMID: 8631588.

53. Palamar M, Shields CL, Marr BP, Eagle RC, Jr., Shields JA. Retinal pigment epithelial tumor in a young Asian female. Eur J Ophthalmol. 2009;19(3):487-9. Epub 2009/04/28. PubMed PMID: 19396802.

54. Palamar M, Thangappan A, Shields CL, Ehya H, Shields JA. Necrotic choroidal melanoma with scleritis and choroidal effusion. Cornea. 2009;28(3):354-6. Epub 2009/04/24. doi: 10.1097/ICO.0b013e3181875463. PubMed PMID: 19387243.

55. Patel K, Sullivan K, Berd D, Mastrangelo MJ, Shields CL, Shields JA, et al. Chemoembolization of the hepatic artery with BCNU for metastatic uveal melanoma: results of a phase II study. Melanoma Res. 2005;15(4):297-304. Epub 2005/07/22. PubMed PMID: 16034309.

56. Phillpotts BA, Sanders RJ, Shields JA, Griffiths JD, Augsburger JA, Shields CL. Uveal melanomas in black patients: a case series and comparative review. J Natl Med Assoc. 1995;87(9):709-14. Epub 1995/09/01. PubMed PMID: 9583969; PubMed Central PMCID: PMC2607893.

57. Potter PD, Shields CL, Shields JA, Flanders AE. The role of magnetic resonance imaging in children with intraocular tumors and simulating lesions. Ophthalmology. 1996;103(11):1774-83. Epub 1996/11/01. PubMed PMID: 8942869.

58. Sagoo MS, Shields CL, Mashayekhi A, Freire J, Emrich J, Reiff J, et al. Plaque radiotherapy for choroidal melanoma encircling the optic disc (circumpapillary choroidal melanoma). Arch Ophthalmol. 2007;125(9):1202-9. Epub 2007/09/12. doi: 10.1001/archopht.125.9.1202. PubMed PMID: 17846359.

59. Sagoo MS, Shields CL, Mashayekhi A, Freire J, Emrich J, Reiff J, et al. Plaque radiotherapy for juxtapapillary choroidal melanoma overhanging the optic disc in 141 consecutive patients. Arch Ophthalmol. 2008;126(11):1515-22. Epub 2008/11/13. doi: 10.1001/archopht.126.11.1515. PubMed PMID: 19001218.

60. Sagoo MS, Shields CL, Mashayekhi A, Freire J, Emrich J, Reiff J, et al. Plaque radiotherapy for juxtapapillary choroidal melanoma: tumor control in 650 consecutive cases. Ophthalmology. 2011;118(2):402-7. Epub 2010/09/04. doi: 10.1016/j.ophtha.2010.06.007. PubMed PMID: 20813410.

61. Saornil MA, Fisher MR, Campbell RJ, Robertson DM, Earle JD, Eagle RC, Jr., et al. Histopathologic study of eyes after iodine I 125 episcleral plaque irradiation for uveal melanoma. Arch Ophthalmol. 1997;115(11):1395-400. Epub 1997/11/21. PubMed PMID: 9366669.

62. Sarici AM, Shah SU, Shields CL, Birdsong RH, Shields JA. Cutaneous halo nevi following plaque radiotherapy for uveal melanoma. Arch Ophthalmol. 2011;129(11):1499-501. Epub 2011/11/16. doi: 10.1001/archophthalmol.2011.313. PubMed PMID: 22084224.

63. Sato T, Babazono A, Shields JA, Shields CL, De Potter P, Mastrangelo MJ. Time to systemic metastases in patients with posterior uveal melanoma. Cancer Invest. 1997;15(2):98-105. Epub 1997/01/01. PubMed PMID: 9095204.

64. Sato T, Eschelman DJ, Gonsalves CF, Terai M, Chervoneva I, McCue PA, et al. Immunoembolization of malignant liver tumors, including uveal melanoma, using granulocyte-macrophage colony-stimulating factor. J Clin Oncol. 2008;26(33):5436-42. Epub 2008/10/08. doi: 10.1200/jco.2008.16.0705. PubMed PMID: 18838710.

65. Shah CP, Weis E, Lajous M, Shields JA, Shields CL. Intermittent and chronic ultraviolet light exposure and uveal melanoma: a meta-analysis. Ophthalmology. 2005;112(9):1599-607. Epub 2005/07/30. doi: 10.1016/j.ophtha.2005.04.020. PubMed PMID: 16051363.

66. Shields CL, Bianciotto C, Pirondini C, Materin MA, Harmon SA, Shields JA. Autofluorescence of orange pigment overlying small choroidal melanoma. Retina. 2007;27(8):1107-11. Epub 2007/11/28. doi: 10.1097/IAE.0b013e31814934ef. PubMed PMID: 18040254.

67. Shields CL, Bianciotto C, Pirondini C, Materin MA, Harmon SA, Shields JA. Autofluorescence of choroidal melanoma in 51 cases. Br J Ophthalmol. 2008;92(5):617-22. Epub 2008/04/29. doi: 10.1136/bjo.2007.130286. PubMed PMID: 18441171.

68. Shields CL, Bianciotto C, Rudich D, Materin MA, Ganguly A, Shields JA. Regression of uveal melanoma after plaque radiotherapy and thermotherapy based on chromosome 3 status. Retina. 2008;28(9):1289-95. Epub 2008/07/17. doi: 10.1097/IAE.0b013e31817f7b3e. PubMed PMID: 18628721.

69. Shields CL, Cater J, Shields JA, Chao A, Krema H, Materin M, et al. Combined plaque radiotherapy and transpupillary thermotherapy for choroidal melanoma: tumor control and treatment complications in 270 consecutive patients. Arch Ophthalmol. 2002;120(7):933-40. Epub 2002/07/06. PubMed PMID: 12096964.

70. Shields CL, Demirci H, Dai V, Marr BP, Mashayekhi A, Materin MA, et al. Intravitreal triamcinolone acetonide for radiation maculopathy after plaque radiotherapy for choroidal melanoma. Retina. 2005;25(7):868-74. Epub 2005/10/06. PubMed PMID: 16205566.

71. Shields CL, Demirci H, Marr BP, Mashayekhi A, Dai VV, Materin MA, et al. Intravitreal triamcinolone acetonide for acute radiation papillopathy. Retina. 2006;26(5):537-44. Epub 2006/06/14. PubMed PMID: 16770260.

72. Shields CL, Demirci H, Materin MA, Marr BP, Mashayekhi A, Shields JA. Clinical factors in the identification of small choroidal melanoma. Can J Ophthalmol. 2004;39(4):351-7. Epub 2004/08/26. PubMed PMID: 15327099.

73. Shields CL, Furuta M, Thangappan A, Nagori S, Mashayekhi A, Lally DR, et al. Metastasis of uveal melanoma millimeter-by-millimeter in 8033 consecutive eyes. Arch Ophthalmol. 2009;127(8):989-98. Epub 2009/08/12. doi: 10.1001/archophthalmol.2009.208. PubMed PMID: 19667335.

74. Shields CL, Ganguly A, Bianciotto CG, Turaka K, Tavallali A, Shields JA. Prognosis of uveal melanoma in 500 cases using genetic testing of fine-needle aspiration biopsy specimens. Ophthalmology. 2011;118(2):396-401. Epub 2010/09/28. doi: 10.1016/j.ophtha.2010.05.023. PubMed PMID: 20869116.

75. Shields CL, Ganguly A, Materin MA, Teixeira L, Mashayekhi A, Swanson LA, et al. Chromosome 3 analysis of uveal melanoma using fine-needle aspiration biopsy at the time of plaque radiotherapy in 140 consecutive cases. Trans Am Ophthalmol Soc. 2007;105:43-52; discussion -3. Epub 2008/04/23. PubMed PMID: 18427593; PubMed Central PMCID: PMC2258107.

76. Shields CL, Ganguly A, Materin MA, Teixeira L, Mashayekhi A, Swanson LA, et al. Chromosome 3 analysis of uveal melanoma using fine-needle aspiration biopsy at the time of plaque radiotherapy in 140 consecutive cases: the Deborah Iverson, MD, Lectureship. Arch Ophthalmol. 2007;125(8):1017-24. Epub 2007/08/19. doi: 10.1001/archopht.125.8.1017. PubMed PMID: 17698747.

77. Shields CL, Honavar SG, Shields JA, Cater J, Demirci H. Circumscribed choroidal hemangioma: clinical manifestations and factors predictive of visual outcome in 200 consecutive cases. Ophthalmology. 2001;108(12):2237-48. Epub 2001/12/06. PubMed PMID: 11733265.

78. Shields CL, Kaliki S, Shah SU, Luo W, Furuta M, Shields JA. Iris melanoma: features and prognosis in 317 children and adults. J AAPOS. 2012;16(1):10-6. Epub 2012/03/01. doi: 10.1016/j.jaapos.2011.10.012. PubMed PMID: 22370659.

79. Shields CL, Kligman BE, Suriano M, Viloria V, Iturralde JC, Shields MV, et al. Phacomatosis pigmentovascularis of cesioflammea type in 7 patients: combination of ocular pigmentation (melanocytosis or melanosis) and nevus flammeus with risk for melanoma. Arch Ophthalmol. 2011;129(6):746-50. Epub 2011/06/15. doi: 10.1001/archophthalmol.2011.135. PubMed PMID: 21670341.

80. Shields CL, Mashayekhi A, Ho T, Cater J, Shields JA. Solitary congenital hypertrophy of the retinal pigment epithelium: clinical features and frequency of enlargement in 330 patients. Ophthalmology. 2003;110(10):1968-76. Epub 2003/10/03. doi: 10.1016/s0161-6420(03)00618-3. PubMed PMID: 14522773.

81. Shields CL, Materin MA, Shields JA. Review of optical coherence tomography for intraocular tumors. Curr Opin Ophthalmol. 2005;16(3):141-54. Epub 2005/05/05. PubMed PMID: 15870570.

82. Shields CL, Materin MA, Teixeira L, Mashayekhi A, Ganguly A, Shields JA. Small choroidal melanoma with chromosome 3 monosomy on fine-needle aspiration biopsy. Ophthalmology. 2007;114(10):1919-24. Epub 2007/08/19. doi: 10.1016/j.ophtha.2007.04.054. PubMed PMID: 17698199.

83. Shields CL, Naseripour M, Cater J, Shields JA, Demirci H, Youseff A, et al. Plaque radiotherapy for large posterior uveal melanomas (> or =8-mm thick) in 354 consecutive patients. Ophthalmology. 2002;109(10):1838-49. Epub 2002/10/03. PubMed PMID: 12359604.

84. Shields CL, Qureshi A, Mashayekhi A, Park C, Sinha N, Zolotarev F, et al. Sector (partial) oculo(dermal) melanocytosis in 89 eyes. Ophthalmology. 2011;118(12):2474-9. Epub 2011/10/25. doi: 10.1016/j.ophtha.2011.05.023. PubMed PMID: 22018683.

85. Shields CL, Salazar PF, Mashayekhi A, Shields JA. Peripheral exudative hemorrhagic chorioretinopathy simulating choroidal melanoma in 173 eyes. Ophthalmology. 2009;116(3):529-35. Epub 2009/01/23. doi: 10.1016/j.ophtha.2008.10.015. PubMed PMID: 19157563.

86. Shields CL, Santos MC, Shields JA, Singh AD, Eagle RC, Jr. Extraocular extension of unrecognized choroidal melanoma simulating a primary optic nerve tumor: report of two cases. Ophthalmology. 1999;106(7):1349-52. Epub 1999/07/16. doi: 10.1016/s0161-6420(99)00723-x. PubMed PMID: 10406620.

87. Shields CL, Shields JA. Transpupillary thermotherapy for choroidal melanoma. Curr Opin Ophthalmol. 1999;10(3):197-203. Epub 1999/10/28. PubMed PMID: 10537779.

88. Shields CL, Shields JA. Subretinal hemorrhage from a retinal arterial macroaneurysm simulating a choroidal melanoma. Ophthalmic Surg Lasers. 2001;32(1):86-7. Epub 2001/02/24. PubMed PMID: 11195753.

89. Shields CL, Shields JA. Clinical features of small choroidal melanoma. Curr Opin Ophthalmol. 2002;13(3):135-41. Epub 2002/05/16. PubMed PMID: 12011680.

90. Shields CL, Shields JA. Recent developments in the management of choroidal melanoma. Curr Opin Ophthalmol. 2004;15(3):244-51. Epub 2004/05/01. PubMed PMID: 15118513.

91. Shields CL, Shields JA. Ocular melanoma: relatively rare but requiring respect. Clin Dermatol. 2009;27(1):122-33. Epub 2008/12/20. doi: 10.1016/j.clindermatol.2008.09.010. PubMed PMID: 19095158.

92. Shields CL, Shields JA, Augsburger JJ. Choroidal osteoma. Surv Ophthalmol. 1988;33(1):17-27. Epub 1988/07/01. PubMed PMID: 3051466.

93. Shields CL, Shields JA, Cater J, Gunduz K, Miyamoto C, Micaily B, et al. Plaque radiotherapy for uveal melanoma: long-term visual outcome in 1106 consecutive patients. Arch Ophthalmol. 2000;118(9):1219-28. Epub 2000/09/12. PubMed PMID: 10980767.

94. Shields CL, Shields JA, Cater J, Lois N, Edelstein C, Gunduz K, et al. Transpupillary thermotherapy for choroidal melanoma: tumor control and visual results in 100 consecutive cases. Ophthalmology. 1998;105(4):581-90. Epub 1998/04/17. doi: 10.1016/s0161-6420(98)94008-8. PubMed PMID: 9544628.

95. Shields CL, Shields JA, De Potter P. Patterns of indocyanine green videoangiography of choroidal tumours. Br J Ophthalmol. 1995;79(3):237-45. Epub 1995/03/01. PubMed PMID: 7703202; PubMed Central PMCID: PMC505071.

96. Shields CL, Shields JA, De Potter P, Cater J, Tardio D, Barrett J. Diffuse choroidal melanoma. Clinical features predictive of metastasis. Arch Ophthalmol. 1996;114(8):956-63. Epub 1996/08/01. PubMed PMID: 8694731.

97. Shields CL, Shields JA, De Potter P, Singh AD. Lack of complications of the hydroxyapatite orbital implant in 250 consecutive cases. Trans Am Ophthalmol Soc. 1993;91:177-89; discussion 89-95. Epub 1993/01/01. PubMed PMID: 8140690; PubMed Central PMCID: PMC1298465.

98. Shields CL, Shields JA, De Potter P, Singh AD. Problems with the hydroxyapatite orbital implant: experience with 250 consecutive cases. Br J Ophthalmol. 1994;78(9):702-6. Epub 1994/09/01. PubMed PMID: 7947552; PubMed Central PMCID: PMC504910.

99. Shields CL, Shields JA, De Potter P, Singh AD, Hernandez C, Brady LW. Treatment of non-resectable malignant iris tumours with custom designed plaque radiotherapy. Br J Ophthalmol. 1995;79(4):306-12. Epub 1995/04/01. PubMed PMID: 7742272; PubMed Central PMCID: PMC505090.

100. Shields CL, Shields JA, DePotter P, Kheterpal S. Transpupillary thermotherapy in the management of choroidal melanoma. Ophthalmology. 1996;103(10):1642-50. Epub 1996/10/01. PubMed PMID: 8874438.

101. Shields CL, Shields JA, Eagle RC, Jr., Cangemi F. Progressive enlargement of acquired retinal astrocytoma in 2 cases. Ophthalmology. 2004;111(2):363-8. Epub 2004/03/17. doi: 10.1016/j.ophtha.2003.05.009. PubMed PMID: 15019391.

102. Shields CL, Shields JA, Eagle RC, Jr., De Potter P. Histopathologic evidence of fibrovascular ingrowth four weeks after placement of the hydroxyapatite orbital implant. Am J Ophthalmol. 1991;111(3):363-6. Epub 1991/03/25. PubMed PMID: 1848039.

103. Shields CL, Shields JA, Eagle RC, Jr., De Potter P, Menduke H. Uveal melanoma and pregnancy. A report of 16 cases. Ophthalmology. 1991;98(11):1667-73. Epub 1991/11/11. PubMed PMID: 1800927.

104. Shields CL, Shields JA, Gunduz K, Freire JE, Mercado G. Radiation therapy for uveal malignant melanoma. Ophthalmic Surg Lasers. 1998;29(5):397-409. Epub 1998/05/26. PubMed PMID: 9599365.

105. Shields CL, Shields JA, Karlsson U, Markoe AM, Brady LW. Reasons for enucleation after plaque radiotherapy for posterior uveal melanoma. Clinical findings. Ophthalmology. 1989;96(6):919-23; discussion 24. Epub 1989/06/01. PubMed PMID: 2740084.

106. Shields CL, Shields JA, Karlsson U, Menduke H, Brady LW. Enucleation after plaque radiotherapy for posterior uveal melanoma. Histopathologic findings. Ophthalmology. 1990;97(12):1665-70. Epub 1990/12/01. PubMed PMID: 2087297.

107. Shields CL, Shields JA, Milite J, De Potter P, Sabbagh R, Menduke H. Uveal melanoma in teenagers and children. A report of 40 cases. Ophthalmology. 1991;98(11):1662-6. Epub 1991/11/01. PubMed PMID: 1800926.

108. Shields CL, Shields JA, Peggs M. Tumors metastatic to the orbit. Ophthal Plast Reconstr Surg. 1988;4(2):73-80. Epub 1988/01/01. PubMed PMID: 3154725.

109. Shields CL, Shields JA, Perez N, Singh AD, Cater J. Primary transpupillary thermotherapy for small choroidal melanoma in 256 consecutive cases: outcomes and limitations. Ophthalmology. 2002;109(2):225-34. Epub 2002/02/05. PubMed PMID: 11825800.

110. Shields CL, Shields JA, Santos MC, Gunduz K, Singh AD, Othmane I. Incomplete spontaneous regression of choroidal melanoma associated with inflammation. Arch Ophthalmol. 1999;117(9):1245-7. Epub 1999/09/25. PubMed PMID: 10496402.

111. Shields CL, Shields JA, Shields MB, Augsburger JJ. Prevalence and mechanisms of secondary intraocular pressure elevation in eyes with intraocular tumors. Ophthalmology. 1987;94(7):839-46. Epub 1987/07/01. PubMed PMID: 3658352.

112. Shields CL, Shields JA, Yarian DL, Augsburger JJ. Intracranial extension of choroidal melanoma via the optic nerve. Br J Ophthalmol. 1987;71(3):172-6. Epub 1987/03/01. PubMed PMID: 3828270; PubMed Central PMCID: PMC1041113.

113. Shields CL, Uysal Y, Marr BP, Lally SE, Rodriques E, Kharod B, et al. Experience with the polymer-coated hydroxyapatite implant after enucleation in 126 patients. Ophthalmology. 2007;114(2):367-73. Epub 2007/02/03. doi: 10.1016/j.ophtha.2006.08.030. PubMed PMID: 17270685.

114. Shields JA, Eagle RC, Jr., Barr CC, Shields CL, Jones DE. Adenocarcinoma of retinal pigment epithelium arising from a juxtapapillary histoplasmosis scar. Arch Ophthalmol. 1994;112(5):650-3. Epub 1994/05/01. PubMed PMID: 8185523.

115. Shields JA, Eagle RC, Jr., Shields CL, Singh AD, Sarin LK. Diffuse uveal melanoma presenting as an amelanotic cystic epibulbar lesion. Retina. 2001;21(5):550-3. Epub 2001/10/20. PubMed PMID: 11642395.

116. Shields JA, Font RL, Eagle RC, Jr., Shields CL, Gass JD. Melanotic schwannoma of the choroid. Immunohistochemistry and electron microscopic observations. Ophthalmology. 1994;101(5):843-9. Epub 1994/05/01. PubMed PMID: 8190469.

117. Shields JA, Mashayekhi A, Ra S, Shields CL. Pseudomelanomas of the posterior uveal tract: the 2006 Taylor R. Smith Lecture. Retina. 2005;25(6):767-71. Epub 2005/09/06. PubMed PMID: 16141866.

118. Shields JA, Materin M, Shields CL, Eagle RC, Jr. Adenoma of the retinal pigment epithelium simulating a juxtapapillary choroidal neovascular membrane. Arch Ophthalmol. 2001;119(2):289-92. Epub 2001/02/15. PubMed PMID: 11176996.

119. Shields JA, Narsipur SS, Shields CL, Sperber DE. Choroidal melanoma in an immunosuppressed child with minimal change nephrotic syndrome. Retina. 2004;24(3):454-5. Epub 2004/06/10. PubMed PMID: 15187671.

120. Shields JA, Perez N, Shields CL, Singh AD, Eagle RC, Jr. Orbital melanoma metastatic from contralateral choroid: management by complete surgical resection. Ophthalmic Surg Lasers. 2002;33(5):416-20. Epub 2002/10/03. PubMed PMID: 12358296.

121. Shields JA, Shields CL. Observations on intraocular leiomyomas. Trans Pa Acad Ophthalmol Otolaryngol. 1990;42:945-50. Epub 1990/01/01. PubMed PMID: 2084991.

122. Shields JA, Shields CL. Massive orbital extension of posterior uveal melanomas. Ophthal Plast Reconstr Surg. 1991;7(4):238-51. Epub 1991/01/01. PubMed PMID: 1764421.

123. Shields JA, Shields CL. Current management of posterior uveal melanoma. Mayo Clin Proc. 1993;68(12):1196-200. Epub 1993/12/01. PubMed PMID: 8246622.

124. Shields JA, Shields CL. CME review: sclerochoroidal calcification: the 2001 Harold Gifford Lecture. Retina. 2002;22(3):251-61. Epub 2002/06/11. PubMed PMID: 12055456.

125. Shields JA, Shields CL, Augsburger JJ, Negrey JN, Jr. Solitary metastasis of choroidal melanoma to the contralateral eyelid. Ophthal Plast Reconstr Surg. 1987;3(1):9-12. Epub 1987/01/01. PubMed PMID: 3154577.

126. Shields JA, Shields CL, Brown GC, Eagle RC, Jr. Mushroom-shaped choroidal metastasis simulating a choroidal melanoma. Retina. 2002;22(6):810-3. Epub 2002/12/12. PubMed PMID: 12476115.

127. Shields JA, Shields CL, De Potter P. Approach to counseling patients with posterior uveal melanoma. Int Ophthalmol Clin. 1993;33(3):143-5. Epub 1993/01/01. PubMed PMID: 8407179.

128. Shields JA, Shields CL, De Potter P, Cu-Unjieng A, Hernandez C, Brady LW. Plaque radiotherapy for uveal melanoma. Int Ophthalmol Clin. 1993;33(3):129-35. Epub 1993/01/01. PubMed PMID: 8407177.

129. Shields JA, Shields CL, De Potter P, Singh AD. Diagnosis and treatment of uveal melanoma. Semin Oncol. 1996;23(6):763-7. Epub 1996/12/01. PubMed PMID: 8970600.

130. Shields JA, Shields CL, Demirci H, Honavar SG, Singh AD. Experience with eyelid-sparing orbital exenteration: the 2000 Tullos O. Coston Lecture. Ophthal Plast Reconstr Surg. 2001;17(5):355-61. Epub 2001/10/20. PubMed PMID: 11642492.

131. Shields JA, Shields CL, DePotter P. Residual intrascleral and intraretinal melanoma. A concern with lamellar sclerouvectomy for uveal melanoma. Am J Ophthalmol. 1992;113(4):464-7. Epub 1992/04/15. PubMed PMID: 1622507.

132. Shields JA, Shields CL, Donoso LA. Management of posterior uveal melanoma. Surv Ophthalmol. 1991;36(3):161-95. Epub 1991/11/01. PubMed PMID: 1776122.

133. Shields JA, Shields CL, Eagle RC, Jr. Mesectodermal leiomyoma of the ciliary body managed by partial lamellar iridocyclochoroidectomy. Ophthalmology. 1989;96(9):1369-76. Epub 1989/09/01. PubMed PMID: 2674827.

134. Shields JA, Shields CL, Eagle RC, Jr., De Potter P. Observations on seven cases of intraocular leiomyoma. The 1993 Byron Demorest Lecture. Arch Ophthalmol. 1994;112(4):521-8. Epub 1994/04/01. PubMed PMID: 8155052.

135. Shields JA, Shields CL, Ehya H, Eagle RC, Jr., De Potter P. Fine-needle aspiration biopsy of suspected intraocular tumors. The 1992 Urwick Lecture. Ophthalmology. 1993;100(11):1677-84. Epub 1993/11/01. PubMed PMID: 8233394.

136. Shields JA, Shields CL, Gunduz K. Visual preservation 18 years after cobalt plaque treatment of choroidal melanoma. Eye (Lond). 1999;13 ( Pt 2):259-60. Epub 1999/08/18. doi: 10.1038/eye.1999.63. PubMed PMID: 10450394.

137. Shields JA, Shields CL, Gunduz K, Eagle RC, Jr. Neoplasms of the retinal pigment epithelium: the 1998 Albert Ruedemann, Sr, memorial lecture, Part 2. Arch Ophthalmol. 1999;117(5):601-8. Epub 1999/05/18. PubMed PMID: 10326956.

138. Shields JA, Shields CL, Kimmel AS, Eagle RC, Jr. Contralateral blindness from chiasmal extension of unsuspected choroidal melanoma. Ophthal Plast Reconstr Surg. 2004;20(5):384-7. Epub 2004/09/21. PubMed PMID: 15377908.

139. Shields JA, Shields CL, Lally SE, Eagle RC, Jr. Harvesting fresh tumor tissue from enucleated eyes: the 2008 Jack S. Guyton lecture. Arch Ophthalmol. 2010;128(2):241-3. Epub 2010/02/10. doi: 10.1001/archophthalmol.2009.396. PubMed PMID: 20142549.

140. Shields JA, Shields CL, Materin M, Sato T, Ganguly A. Role of cytogenetics in management of uveal melanoma. Arch Ophthalmol. 2008;126(3):416-9. Epub 2008/03/12. doi: 10.1001/archopht.126.3.416. PubMed PMID: 18332325.

141. Shields JA, Shields CL, Naseripor M, Eagle RC, Miller J. Choroidal melanoma in a black patient with oculodermal melanocytosis. Retina. 2002;22(1):126-8. Epub 2002/03/09. PubMed PMID: 11884898.

142. Shields JA, Shields CL, Scartozzi R. Survey of 1264 patients with orbital tumors and simulating lesions: The 2002 Montgomery Lecture, part 1. Ophthalmology. 2004;111(5):997-1008. Epub 2004/05/04. doi: 10.1016/j.ophtha.2003.01.002. PubMed PMID: 15121380.

143. Shields JA, Shields CL, Shah P, Sivalingam V. Partial lamellar sclerouvectomy for ciliary body and choroidal tumors. Ophthalmology. 1991;98(6):971-83. Epub 1991/06/01. PubMed PMID: 1866153.

144. Shields JA, Shields CL, Shakin EP, Kobetz LE. Metastasis of choroidal melanoma to the contralateral choroid, orbit, and eyelid. Br J Ophthalmol. 1988;72(6):456-60. Epub 1988/06/01. PubMed PMID: 3390423; PubMed Central PMCID: PMC1041482.

145. Shields JA, Shields CL, Slakter J, Wood W, Yannuzzi LA. Locally invasive tumors arising from hyperplasia of the retinal pigment epithelium. Retina. 2001;21(5):487-92. Epub 2001/10/20. PubMed PMID: 11642378.

146. Shields JA, Shields CL, Suvarnamani C, Tantisira M, Shah P. Orbital exenteration with eyelid sparing: indications, technique, and results. Ophthalmic Surg. 1991;22(5):292-7. Epub 1991/05/01. PubMed PMID: 1852385.

147. Shields JA, Stephens RF, Eagle RC, Jr., Shields CL, De Potter P. Progressive enlargement of a circumscribed choroidal hemangioma. A clinicopathologic correlation. Arch Ophthalmol. 1992;110(9):1276-8. Epub 1992/09/11. PubMed PMID: 1520116.

148. Singh AD, Boghosian-Sell L, Wary KK, Shields CL, De Potter P, Donoso LA, et al. Cytogenetic findings in primary uveal melanoma. Cancer Genet Cytogenet. 1994;72(2):109-15. Epub 1994/02/01. PubMed PMID: 8143268.

149. Singh AD, Croce CM, Wary KK, Shields JA, Donoso LA, Shields CL, et al. Familial uveal melanoma: absence of germline mutations involving the cyclin-dependent kinase-4 inhibitor gene (p16). Ophthalmic Genet. 1996;17(1):39-40. Epub 1996/03/01. PubMed PMID: 8740697.

150. Singh AD, De Potter P, Fijal BA, Shields CL, Shields JA, Elston RC. Lifetime prevalence of uveal melanoma in white patients with oculo(dermal) melanocytosis. Ophthalmology. 1998;105(1):195-8. Epub 1998/01/27. PubMed PMID: 9442799.

151. Singh AD, Demirci H, Shields CL, Shields JA, Smith AF. Concurrent choroidal melanoma in son and father. Am J Ophthalmol. 2000;130(5):679-80. Epub 2000/11/18. PubMed PMID: 11078855.

152. Singh AD, Donoso LA, Jackson L, Shields CL, De Potter P, Shields JA. Familial uveal melanoma: absence of constitutional cytogenic abnormalities in 14 cases. Arch Ophthalmol. 1996;114(4):502-3. Epub 1996/04/01. PubMed PMID: 8602802.

153. Singh AD, Eagle RC, Jr., Shields CL, Shields JA. Clinicopathologic reports, case reports, and small case series: enucleation following transpupillary thermotherapy of choroidal melanoma: clinicopathologic correlations. Arch Ophthalmol. 2003;121(3):397-400. Epub 2003/03/06. PubMed PMID: 12617712.

154. Singh AD, Shields CL, De Potter P, Shields JA. Tear of the retinal pigment epithelium after brachytherapy of choroidal melanoma. Retina. 1994;14(1):81-2. Epub 1994/01/01. PubMed PMID: 8016468.

155. Singh AD, Shields CL, De Potter P, Shields JA, Trock B, Cater J, et al. Familial uveal melanoma. Clinical observations on 56 patients. Arch Ophthalmol. 1996;114(4):392-9. Epub 1996/04/01. PubMed PMID: 8602775.

156. Singh AD, Shields CL, Shields JA. Prognostic factors in uveal melanoma. Melanoma Res. 2001;11(3):255-63. Epub 2001/07/27. PubMed PMID: 11468514.

157. Singh AD, Shields CL, Shields JA, De Potter P. Bilateral primary uveal melanoma. Bad luck or bad genes? Ophthalmology. 1996;103(2):256-62. Epub 1996/02/01. PubMed PMID: 8594511.

158. Singh AD, Shields CL, Shields JA, Eagle RC, De Potter P. Uveal melanoma and familial atypical mole and melanoma (FAM-M) syndrome. Ophthalmic Genet. 1995;16(2):53-61. Epub 1995/06/01. PubMed PMID: 7493157.

159. Singh AD, Shields CL, Shields JA, Sato T. Uveal melanoma in young patients. Arch Ophthalmol. 2000;118(7):918-23. Epub 2000/07/19. PubMed PMID: 10900104.

160. Singh AD, Shields CL, Shields JA, Sternberg P, Jr. Occurrence of retinoblastoma and uveal melanoma in the same patient. Retina. 2000;20(3):305-6. Epub 2000/06/29. PubMed PMID: 10872940.

161. Singh AD, Shields JA, Eagle RC, Shields CL, Marmor M, De Potter P. Iris melanoma in a ten-year-old boy with familial atypical mole-melanoma (FAM-M) syndrome. Ophthalmic Genet. 1994;15(3-4):145-9. Epub 1994/09/01. PubMed PMID: 7749669.

162. Singh AD, Shields JA, Shields CL, Sato T. Choroidal melanoma metastatic to the contralateral choroid. Am J Ophthalmol. 2001;132(6):941-3. Epub 2001/12/04. PubMed PMID: 11730671.

163. Singh AD, Wang MX, Donoso LA, Shields CL, De Potter P, Shields JA. Genetic aspects of uveal melanoma: a brief review. Semin Oncol. 1996;23(6):768-72. Epub 1996/12/01. PubMed PMID: 8970601.

164. Singh AD, Wang MX, Donoso LA, Shields CL, Potter PD, Shields JA, et al. Familial uveal melanoma, III. Is the occurrence of familial uveal melanoma coincidental? Arch Ophthalmol. 1996;114(9):1101-4. Epub 1996/09/01. PubMed PMID: 8790096.

165. Turaka K, Shields CL, Shah CP, Say EA, Shields JA. Bilateral uveal melanoma in an arc welder. Graefes Arch Clin Exp Ophthalmol. 2011;249(1):141-4. Epub 2010/09/21. doi: 10.1007/s00417-010-1516-5. PubMed PMID: 20853114.

166. Weis E, Shah CP, Lajous M, Shields JA, Shields CL. The association between host susceptibility factors and uveal melanoma: a meta-analysis. Arch Ophthalmol. 2006;124(1):54-60. Epub 2006/01/13. doi: 10.1001/archopht.124.1.54. PubMed PMID: 16401785.

167. Weis E, Shah CP, Lajous M, Shields JA, Shields CL. The association of cutaneous and iris nevi with uveal melanoma: a meta-analysis. Ophthalmology. 2009;116(3):536-43 e2. Epub 2009/01/27. doi: 10.1016/j.ophtha.2008.10.008. PubMed PMID: 19167086.

168. Yamamoto A, Chervoneva I, Sullivan KL, Eschelman DJ, Gonsalves CF, Mastrangelo MJ, et al. High-dose immunoembolization: survival benefit in patients with hepatic metastases from uveal melanoma. Radiology. 2009;252(1):290-8. Epub 2009/06/30. doi: 10.1148/radiol.2521081252. PubMed PMID: 19561263.

169. Zhao DY, Gunduz K, Shields CL, Shields JA. Choroidal melanoma-associated retinal and retinal pigment epithelial changes. J Ophthalmic Nurs Technol. 1998;17(3):110-4. Epub 1998/07/25. PubMed PMID: 9677983.

170. Zhao DY, Shields CL, Shields JA, Gunduz K. Update on the management of posterior uveal melanoma. J Ophthalmic Nurs Technol. 1998;17(2):66-71. Epub 1998/07/25. PubMed PMID: 9677975.

171. Ah-Fat FG, Damato BE. Delays in the diagnosis of uveal melanoma and effect on treatment. Eye (Lond). 1998;12 ( Pt 5):781-2. Epub 1999/03/10. doi: 10.1038/eye.1998.202. PubMed PMID: 10070508.

172. Anderson MF, Callejo S, Bridson JD, Damato BE. Diagnostic information provided by referrers to patients with suspected uveal melanoma. Eye (Lond). 2011;25(3):392. Epub 2010/12/18. doi: 10.1038/eye.2010.194. PubMed PMID: 21164532; PubMed Central PMCID: PMC3178319.

173. Angi M, Damato B, Kalirai H, Dodson A, Taktak A, Coupland SE. Immunohistochemical assessment of mitotic count in uveal melanoma. Acta Ophthalmol. 2011;89(2):e155-60. Epub 2009/11/11. doi: 10.1111/j.1755-3768.2009.01769.x. PubMed PMID: 19900200.

174. Bechrakis NE, Sehu KW, Lee WR, Damato BE, Foerster MH. Transformation of cell type in uveal melanomas: a quantitative histologic analysis. Arch Ophthalmol. 2000;118(10):1406-12. Epub 2000/10/13. PubMed PMID: 11030824.

175. Borthwick NJ, Thombs J, Polak M, Gabriel FG, Hungerford JL, Damato B, et al. The biology of micrometastases from uveal melanoma. J Clin Pathol. 2011;64(8):666-71. Epub 2011/05/20. doi: 10.1136/jcp.2010.087999. PubMed PMID: 21593344.

176. Bowling BS, Damato BE, Foy PM. Choroidal melanoma metastatic to the contralateral orbit: implications for patient management. Eye (Lond). 1994;8 ( Pt 1):144-5. Epub 1994/01/01. doi: 10.1038/eye.1994.29. PubMed PMID: 8013710.

177. Bowyer J, Jones A, Damato B, Leach A, Shankar J, Tripathi A. The subjective experience of early postoperative pain following retrobulbar anaesthesia for enucleation and primary orbital implant. Orbit. 2003;22(4):271-7. Epub 2003/12/20. PubMed PMID: 14685901.

178. Brandberg Y, Damato B, Kivela T, Kock E, Seregard S. The EORTC ophthalmic oncology quality of life questionnaire module (EORTC QLQ-OPT30). Development and pre-testing (Phase I-III). Eye (Lond). 2004;18(3):283-9. Epub 2004/03/09. doi: 10.1038/sj.eye.6700639. PubMed PMID: 15004578.

179. Calipel A, Abonnet V, Nicole O, Mascarelli F, Coupland SE, Damato B, et al. Status of RASSF1A in uveal melanocytes and melanoma cells. Mol Cancer Res. 2011;9(9):1187-98. Epub 2011/07/27. doi: 10.1158/1541-7786.mcr-10-0437. PubMed PMID: 21788308.

180. Callejo SA, Dopierala J, Coupland SE, Damato B. Sudden growth of a choroidal melanoma and multiplex ligation-dependent probe amplification findings suggesting late transformation to monosomy 3 type. Arch Ophthalmol. 2011;129(7):958-60. Epub 2011/07/13. doi: 10.1001/archophthalmol.2011.181. PubMed PMID: 21746991.

181. Carbonaro F, Damato B. 'I took a gamble and I lost': unwarranted patient regrets about choice of treatment of uveal melanoma. Eye (Lond). 2005;19(5):594-5. Epub 2004/06/09. doi: 10.1038/sj.eye.6701532. PubMed PMID: 15184936.

182. Cochran AJ, Foulds WS, Damato BE, Trope GE, Morrison L, Lee WR. Assessment of immunological techniques in the diagnosis and prognosis of ocular malignant melanoma. Br J Ophthalmol. 1985;69(3):171-6. Epub 1985/03/01. PubMed PMID: 3884037; PubMed Central PMCID: PMC1040556.

183. Cook SA, Damato B, Marshall E, Salmon P. Psychological aspects of cytogenetic testing of uveal melanoma: preliminary findings and directions for future research. Eye (Lond). 2009;23(3):581-5. Epub 2008/03/18. doi: 10.1038/eye.2008.54. PubMed PMID: 18344957.

184. Cook SA, Damato B, Marshall E, Salmon P. Reconciling the principle of patient autonomy with the practice of informed consent: decision-making about prognostication in uveal melanoma. Health Expect. 2011;14(4):383-96. Epub 2010/10/30. doi: 10.1111/j.1369-7625.2010.00639.x. PubMed PMID: 21029286.

185. Coupland SE, Campbell I, Damato B. Routes of extraocular extension of uveal melanoma: risk factors and influence on survival probability. Ophthalmology. 2008;115(10):1778-85. Epub 2008/06/17. doi: 10.1016/j.ophtha.2008.04.025. PubMed PMID: 18554722.

186. Coupland SE, Vorum H, Mandal N, Kalirai H, Honore B, Urbak SF, et al. Proteomics of uveal melanomas suggests HSP-27 as a possible surrogate marker of chromosome 3 loss. Invest Ophthalmol Vis Sci. 2010;51(1):12-20. Epub 2009/08/01. doi: 10.1167/iovs.09-3913. PubMed PMID: 19643972.

187. Cryan LM, Paraoan L, Hiscott P, Damato BE, Grierson I, Gray D, et al. Expression of COX-2 and prognostic outcome in uveal melanoma. Curr Eye Res. 2008;33(2):177-84. Epub 2008/02/23. doi: 10.1080/02713680701867908. PubMed PMID: 18293189.

188. Damato B. Adjunctive plaque radiotherapy after local resection of uveal melanoma. Front Radiat Ther Oncol. 1997;30:123-32. Epub 1997/01/01. PubMed PMID: 9205892.

189. Damato B. Disease and "dis-ease" in patients with uveal melanoma. Br J Ophthalmol. 2000;84(4):343-4. Epub 2000/03/24. PubMed PMID: 10729287; PubMed Central PMCID: PMC1723436.

190. Damato B. Detection of uveal melanoma by optometrists in the United Kingdom. Ophthalmic Physiol Opt. 2001;21(4):268-71. Epub 2001/06/30. PubMed PMID: 11430620.

191. Damato B. Time to treatment of uveal melanoma in the United Kingdom. Eye (Lond). 2001;15(Pt 2):155-8. Epub 2001/05/08. doi: 10.1038/eye.2001.51. PubMed PMID: 11339581.

192. Damato B. Developments in the management of uveal melanoma. Clin Experiment Ophthalmol. 2004;32(6):639-47. Epub 2004/12/04. doi: 10.1111/j.1442-9071.2004.00917.x. PubMed PMID: 15575836.

193. Damato B. The role of eyewall resection in uveal melanoma management. Int Ophthalmol Clin. 2006;46(1):81-93. Epub 2005/12/21. PubMed PMID: 16365557.

194. Damato B. Choroidal melanoma endoresection, dandelions and allegory-based medicine. Br J Ophthalmol. 2008;92(8):1013-4. Epub 2008/07/26. doi: 10.1136/bjo.2008.143214. PubMed PMID: 18653590.

195. Damato B. Does ocular treatment of uveal melanoma influence survival? Br J Cancer. 2010;103(3):285-90. Epub 2010/07/28. doi: 10.1038/sj.bjc.6605765. PubMed PMID: 20661247; PubMed Central PMCID: PMC2920019.

196. Damato B, Coupland SE. A reappraisal of the significance of largest basal diameter of posterior uveal melanoma. Eye (Lond). 2009;23(12):2152-60; quiz 61-2. Epub 2009/10/31. doi: 10.1038/eye.2009.235-cme. PubMed PMID: 19876071.

197. Damato B, Coupland SE. Translating uveal melanoma cytogenetics into clinical care. Arch Ophthalmol. 2009;127(4):423-9. Epub 2009/04/15. doi: 10.1001/archophthalmol.2009.40. PubMed PMID: 19365018.

198. Damato B, Coupland SE. Genomic typing of uveal melanoma. Arch Ophthalmol. 2009;127(1):113-4; author reply 4-5. Epub 2009/01/14. doi: 10.1001/archophthalmol.2008.529. PubMed PMID: 19139354.

199. Damato B, Dopierala J, Klaasen A, van Dijk M, Sibbring J, Coupland SE. Multiplex ligation-dependent probe amplification of uveal melanoma: correlation with metastatic death. Invest Ophthalmol Vis Sci. 2009;50(7):3048-55. Epub 2009/02/03. doi: 10.1167/iovs.08-3165. PubMed PMID: 19182252.

200. Damato B, Dopierala JA, Coupland SE. Genotypic profiling of 452 choroidal melanomas with multiplex ligation-dependent probe amplification. Clin Cancer Res. 2010;16(24):6083-92. Epub 2010/10/27. doi: 10.1158/1078-0432.ccr-10-2076. PubMed PMID: 20975103.

201. Damato B, Duke C, Coupland SE, Hiscott P, Smith PA, Campbell I, et al. Cytogenetics of uveal melanoma: a 7-year clinical experience. Ophthalmology. 2007;114(10):1925-31. Epub 2007/08/28. doi: 10.1016/j.ophtha.2007.06.012. PubMed PMID: 17719643.

202. Damato B, Eleuteri A, Fisher AC, Coupland SE, Taktak AF. Artificial neural networks estimating survival probability after treatment of choroidal melanoma. Ophthalmology. 2008;115(9):1598-607. Epub 2008/03/18. doi: 10.1016/j.ophtha.2008.01.032. PubMed PMID: 18342942.

203. Damato B, Eleuteri A, Taktak AF, Coupland SE. Estimating prognosis for survival after treatment of choroidal melanoma. Prog Retin Eye Res. 2011;30(5):285-95. Epub 2011/06/11. doi: 10.1016/j.preteyeres.2011.05.003. PubMed PMID: 21658465.

204. Damato B, Foulds WS. Indications for trans-scleral local resection of uveal melanoma. Br J Ophthalmol. 1996;80(11):1029-30. Epub 1996/11/01. PubMed PMID: 8976740; PubMed Central PMCID: PMC505690.

205. Damato B, Groenewald C, McGalliard J, Wong D. Endoresection of choroidal melanoma. Br J Ophthalmol. 1998;82(3):213-8. Epub 1998/05/29. PubMed PMID: 9602614; PubMed Central PMCID: PMC1722501.

206. Damato B, Groenewald CP, McGalliard JN, Wong D. Rhegmatogenous retinal detachment after transscleral local resection of choroidal melanoma. Ophthalmology. 2002;109(11):2137-43. Epub 2002/11/05. PubMed PMID: 12414429.

207. Damato B, Jones AG. Uveal melanoma: resection techniques. Ophthalmol Clin North Am. 2005;18(1):119-28, ix. Epub 2005/03/15. doi: 10.1016/j.ohc.2004.08.010. PubMed PMID: 15763197.

208. Damato B, Kacperek A, Chopra M, Campbell IR, Errington RD. Proton beam radiotherapy of choroidal melanoma: the Liverpool-Clatterbridge experience. Int J Radiat Oncol Biol Phys. 2005;62(5):1405-11. Epub 2005/07/21. doi: 10.1016/j.ijrobp.2005.01.016. PubMed PMID: 16029800.

209. Damato B, Lecuona K. Conservation of eyes with choroidal melanoma by a multimodality approach to treatment: an audit of 1632 patients. Ophthalmology. 2004;111(5):977-83. Epub 2004/05/04. doi: 10.1016/j.ophtha.2003.09.028. PubMed PMID: 15121377.

210. Damato B, Patel I, Campbell IR, Mayles HM, Errington RD. Visual acuity after Ruthenium(106) brachytherapy of choroidal melanomas. Int J Radiat Oncol Biol Phys. 2005;63(2):392-400. Epub 2005/07/02. doi: 10.1016/j.ijrobp.2005.02.059. PubMed PMID: 15990248.

211. Damato B, Patel I, Campbell IR, Mayles HM, Errington RD. Local tumor control after 106Ru brachytherapy of choroidal melanoma. Int J Radiat Oncol Biol Phys. 2005;63(2):385-91. Epub 2005/05/26. doi: 10.1016/j.ijrobp.2005.02.017. PubMed PMID: 15913907.

212. Damato B, Wong D, Green FD, Mackenzie JM. Intrascleral recurrence of uveal melanoma after transretinal "endoresection". Br J Ophthalmol. 2001;85(1):114-5. Epub 2001/02/24. PubMed PMID: 11201945; PubMed Central PMCID: PMC1723712.

213. Damato BE. An approach to the management of patients with uveal melanoma. Eye (Lond). 1993;7 ( Pt 3):388-97. Epub 1993/01/01. doi: 10.1038/eye.1993.77. PubMed PMID: 8224293.

214. Damato BE. Local resection of uveal melanoma. Bull Soc Belge Ophtalmol. 1993;248:11-7. Epub 1993/01/01. PubMed PMID: 8044325.

215. Damato BE. Local resection of uveal melanoma. Dev Ophthalmol. 2012;49:66-80. Epub 2011/11/02. doi: 10.1159/000328261. PubMed PMID: 22042014.

216. Damato BE. Treatment selection for uveal melanoma. Dev Ophthalmol. 2012;49:16-26. Epub 2011/11/02. doi: 10.1159/000328251. PubMed PMID: 22042010.

217. Damato BE, Campbell AM, McGuire BJ, Lee WR, Foulds WS. Monoclonal antibodies to human primary uveal melanomas demonstrate tumor heterogeneity. Invest Ophthalmol Vis Sci. 1986;27(9):1362-7. Epub 1986/09/01. PubMed PMID: 3744725.

218. Damato BE, Campbell AM, McGuire BJ, Lee WR, Foulds WS. Monoclonal antibodies to uveal melanoma. Eye (Lond). 1987;1 ( Pt 6):686-90. Epub 1987/01/01. doi: 10.1038/eye.1987.112. PubMed PMID: 3454763.

219. Damato BE, Campbell AM, McGuire BJ, Lee WR, Foulds WS. B-lymphocytes from melanoma patients and normal individuals react with melanoma cells but also with irrelevant antigens. Br J Cancer. 1988;58(2):182-5. Epub 1988/08/01. PubMed PMID: 3262365; PubMed Central PMCID: PMC2246765.

220. Damato BE, Coupland SE. Differences in uveal melanomas between men and women from the British Isles. Eye (Lond). 2012;26(2):292-9. Epub 2011/11/15. doi: 10.1038/eye.2011.272. PubMed PMID: 22079972; PubMed Central PMCID: PMC3272186.

221. Damato BE, Paul J, Foulds WS. Predictive factors of visual outcome after local resection of choroidal melanoma. Br J Ophthalmol. 1993;77(10):616-23. Epub 1993/10/01. PubMed PMID: 8218028; PubMed Central PMCID: PMC504601.

222. Damato BE, Paul J, Foulds WS. Risk factors for metastatic uveal melanoma after trans-scleral local resection. Br J Ophthalmol. 1996;80(2):109-16. Epub 1996/02/01. PubMed PMID: 8814739; PubMed Central PMCID: PMC505400.

223. Damato BE, Paul J, Foulds WS. Risk factors for residual and recurrent uveal melanoma after trans-scleral local resection. Br J Ophthalmol. 1996;80(2):102-8. Epub 1996/02/01. PubMed PMID: 8814738; PubMed Central PMCID: PMC505399.

224. Davies L, Gray D, Spiller D, White MR, Damato B, Grierson I, et al. P53 apoptosis mediator PERP: localization, function and caspase activation in uveal melanoma. J Cell Mol Med. 2009;13(8B):1995-2007. Epub 2008/12/02. doi: 10.1111/j.1582-4934.2008.00590.x. PubMed PMID: 19040420.

225. Dopierala J, Damato BE, Lake SL, Taktak AF, Coupland SE. Genetic heterogeneity in uveal melanoma assessed by multiplex ligation-dependent probe amplification. Invest Ophthalmol Vis Sci. 2010;51(10):4898-905. Epub 2010/05/21. doi: 10.1167/iovs.09-5004. PubMed PMID: 20484589.

226. Durie FH, Campbell AM, Lee WR, Damato BE. Analysis of lymphocytic infiltration in uveal melanoma. Invest Ophthalmol Vis Sci. 1990;31(10):2106-10. Epub 1990/10/01. PubMed PMID: 2211008.

227. Durie FH, George WD, Campbell AM, Damato BE. Analysis of clonality of tumour infiltrating lymphocytes in breast cancer and uveal melanoma. Immunol Lett. 1992;33(3):263-9. Epub 1992/08/01. PubMed PMID: 1428001.

228. Foulds WS, Damato BE. Alternatives to enucleation in the management of choroidal melanoma. Aust N Z J Ophthalmol. 1986;14(1):19-27. Epub 1986/02/01. PubMed PMID: 3964476.

229. Foulds WS, Damato BE. Low-energy long-exposure laser therapy in the management of choroidal melanoma. Graefes Arch Clin Exp Ophthalmol. 1986;224(1):26-31. Epub 1986/01/01. PubMed PMID: 3943731.

230. Foulds WS, Damato BE, Burton RL. Local resection versus enucleation in the management of choroidal melanoma. Eye (Lond). 1987;1 ( Pt 6):676-9. Epub 1987/01/01. doi: 10.1038/eye.1987.110. PubMed PMID: 3454761.

231. Guerin E, Hiscott P, Damato B. Choroidal neovascular membrane in a series of cases of malignant melanoma of the choroid. Acta Ophthalmol Scand. 2006;84(3):323-7. Epub 2006/05/18. doi: 10.1111/j.1600-0420.2005.00630.x. PubMed PMID: 16704692.

232. Hadden PW, Damato BE. Simultaneous presentation of choroidal melanoma in mother and daughter. Br J Ophthalmol. 2003;87(6):793-4. Epub 2003/05/29. PubMed PMID: 12770989; PubMed Central PMCID: PMC1771724.

233. Hadden PW, Damato BE. Consecutive choroidal melanoma in the same eye of a patient. Am J Ophthalmol. 2003;135(5):728-9. Epub 2003/04/30. PubMed PMID: 12719093.

234. Hadden PW, Damato BE, McKay IC. Bilateral uveal melanoma: a series of four cases. Eye (Lond). 2003;17(5):613-6. Epub 2003/07/12. doi: 10.1038/sj.eye.6700435. PubMed PMID: 12855969.

235. Hadden PW, Hiscott PS, Damato BE. Histopathology of eyes enucleated after endoresection of choroidal melanoma. Ophthalmology. 2004;111(1):154-60. Epub 2004/01/09. doi: 10.1016/j.ophtha.2003.05.007. PubMed PMID: 14711728.

236. Hearle N, Damato BE, Humphreys J, Wixey J, Green H, Stone J, et al. Contribution of germline mutations in BRCA2, P16(INK4A), P14(ARF) and P15 to uveal melanoma. Invest Ophthalmol Vis Sci. 2003;44(2):458-62. Epub 2003/01/31. PubMed PMID: 12556369.

237. Hearle N, Humphreys J, Damato BE, Wort R, Talaban R, Wixey J, et al. Role of MC1R variants in uveal melanoma. Br J Cancer. 2003;89(10):1961-5. Epub 2003/11/13. doi: 10.1038/sj.bjc.6601358. PubMed PMID: 14612910; PubMed Central PMCID: PMC2394456.

238. Houlston RS, Damato BE. Genetic predisposition to ocular melanoma. Eye (Lond). 1999;13 ( Pt 1):43-6. Epub 1999/07/09. doi: 10.1038/eye.1999.9. PubMed PMID: 10396383.

239. Kalirai H, Damato BE, Coupland SE. Uveal melanoma cell lines contain stem-like cells that self-renew, produce differentiated progeny, and survive chemotherapy. Invest Ophthalmol Vis Sci. 2011;52(11):8458-66. Epub 2011/09/08. doi: 10.1167/iovs.11-7379. PubMed PMID: 21896849.

240. Kent D, Noonan CP, Damato BE. Management of Irish patients with intraocular melanoma referred to Liverpool, England. Acta Ophthalmol Scand. 1998;76(5):584-8. Epub 1998/11/24. PubMed PMID: 9826044.

241. Khan J, Damato BE. Accuracy of choroidal melanoma diagnosis by general ophthalmologists: a prospective study. Eye (Lond). 2007;21(5):595-7. Epub 2006/02/14. doi: 10.1038/sj.eye.6702276. PubMed PMID: 16470216.

242. Kim JW, Damato BE, Hiscott P. Noncontiguous tumor recurrence of posterior uveal melanoma after transscleral local resection. Arch Ophthalmol. 2002;120(12):1659-64. Epub 2002/12/10. PubMed PMID: 12470139.

243. Kivela T, Puusaari I, Damato B. Transscleral resection versus iodine brachytherapy for choroidal malignant melanomas 6 millimeters or more in thickness: a matched case-control study. Ophthalmology. 2003;110(11):2235-44. Epub 2003/11/05. doi: 10.1016/j.ophtha.2003.02.001. PubMed PMID: 14597535.

244. Lake SL, Coupland SE, Taktak AF, Damato BE. Whole-genome microarray detects deletions and loss of heterozygosity of chromosome 3 occurring exclusively in metastasizing uveal melanoma. Invest Ophthalmol Vis Sci. 2010;51(10):4884-91. Epub 2010/05/07. doi: 10.1167/iovs.09-5083. PubMed PMID: 20445121.

245. Lake SL, Damato BE, Dopierala J, Baudo MM, Taktak AF, Coupland SE. Multiplex ligation-dependent probe amplification analysis of uveal melanoma with extraocular extension demonstrates heterogeneity of gross chromosomal abnormalities. Invest Ophthalmol Vis Sci. 2011;52(8):5559-64. Epub 2011/06/11. doi: 10.1167/iovs.10-6845. PubMed PMID: 21659309.

246. Lake SL, Jmor F, Dopierala J, Taktak AF, Coupland SE, Damato BE. Multiplex ligation-dependent probe amplification of conjunctival melanoma reveals common BRAF V600E gene mutation and gene copy number changes. Invest Ophthalmol Vis Sci. 2011;52(8):5598-604. Epub 2011/06/23. doi: 10.1167/iovs.10-6934. PubMed PMID: 21693616.

247. Lake SL, Kalirai H, Dopierala J, Damato BE, Coupland SE. Comparison of formalin-fixed and snap-frozen samples analyzed by multiplex ligation-dependent probe amplification for prognostic testing in uveal melanoma. Invest Ophthalmol Vis Sci. 2012;53(6):2647-52. Epub 2012/03/20. doi: 10.1167/iovs.12-9584. PubMed PMID: 22427594.

248. Omulecki W, Damato BE, Sekundo W, Lee WR, Toczyska-Rozentryt E, Omulecka A. Bilateral uveal melanoma presenting simultaneously. Ger J Ophthalmol. 1994;3(4-5):228-31. Epub 1994/08/01. PubMed PMID: 7804108.

249. Ordonez JL, Paraoan L, Hiscott P, Gray D, Garcia-Finana M, Grierson I, et al. Differential expression of angioregulatory matricellular proteins in posterior uveal melanoma. Melanoma Res. 2005;15(6):495-502. Epub 2005/11/30. PubMed PMID: 16314734.

250. Paraoan L, Gray D, Hiscott P, Ebrahimi B, Damato B, Grierson I. Expression of p53-induced apoptosis effector PERP in primary uveal melanomas: downregulation is associated with aggressive type. Exp Eye Res. 2006;83(4):911-9. Epub 2006/06/21. doi: 10.1016/j.exer.2006.04.016. PubMed PMID: 16784742.

251. Paraoan L, Gray D, Hiscott P, Garcia-Finana M, Lane B, Damato B, et al. Cathepsin S and its inhibitor cystatin C: imbalance in uveal melanoma. Front Biosci. 2009;14:2504-13. Epub 2009/03/11. PubMed PMID: 19273215.

252. Pearce IA, Smith PA, Damato BE. Pseudocystic ultrasound appearance of choroidal melanoma. Br J Ophthalmol. 1997;81(6):517. Epub 1997/06/01. PubMed PMID: 9274424; PubMed Central PMCID: PMC1722208.

253. Pe'er J, Stefani FH, Seregard S, Kivela T, Lommatzsch P, Prause JU, et al. Cell proliferation activity in posterior uveal melanoma after Ru-106 brachytherapy: an EORTC ocular oncology group study. Br J Ophthalmol. 2001;85(10):1208-12. Epub 2001/09/25. PubMed PMID: 11567966; PubMed Central PMCID: PMC1723747.

254. Puusaari I, Damato B, Kivela T. Transscleral local resection versus iodine brachytherapy for uveal melanomas that are large because of tumour height. Graefes Arch Clin Exp Ophthalmol. 2007;245(4):522-33. Epub 2006/11/18. doi: 10.1007/s00417-006-0461-9. PubMed PMID: 17111148.

255. Russo A, Coupland SE, O'Keefe M, Damato BE. Choroidal melanoma in a 7-year-old child treated by trans-scleral local resection. Graefes Arch Clin Exp Ophthalmol. 2010;248(5):747-9. Epub 2010/02/10. doi: 10.1007/s00417-009-1295-z. PubMed PMID: 20143236.

256. Russo A, Coupland SE, Raja V, Damato BE. Recurrent choroidal melanoma after brachytherapy in an eye with scleral calcification. Acta Ophthalmol. 2011;89(8):e657-8. Epub 2010/10/16. doi: 10.1111/j.1755-3768.2010.02019.x. PubMed PMID: 20946331.

257. Schalenbourg A, Coupland S, Kacperek A, Damato B. Iridocyclectomy for neovascular glaucoma caused by proton-beam radiotherapy of pigmented ciliary adenocarcinoma. Graefes Arch Clin Exp Ophthalmol. 2008;246(10):1499-501. Epub 2008/06/27. doi: 10.1007/s00417-008-0852-1. PubMed PMID: 18581131.

258. Scholes AG, Damato BE, Nunn J, Hiscott P, Grierson I, Field JK. Monosomy 3 in uveal melanoma: correlation with clinical and histologic predictors of survival. Invest Ophthalmol Vis Sci. 2003;44(3):1008-11. Epub 2003/02/26. PubMed PMID: 12601021.

259. Scholes AG, Hagan S, Hiscott P, Damato BE, Grierson I. Overexpression of epidermal growth factor receptor restricted to macrophages in uveal melanoma. Arch Ophthalmol. 2001;119(3):373-7. Epub 2001/03/20. PubMed PMID: 11231770.

260. Scholes AG, Liloglou T, Maloney P, Hagan S, Nunn J, Hiscott P, et al. Loss of heterozygosity on chromosomes 3, 9, 13, and 17, including the retinoblastoma locus, in uveal melanoma. Invest Ophthalmol Vis Sci. 2001;42(11):2472-7. Epub 2001/10/03. PubMed PMID: 11581185.

261. Singh AD, Damato B, Howard P, Harbour JW. Uveal melanoma: genetic aspects. Ophthalmol Clin North Am. 2005;18(1):85-97, viii. Epub 2005/03/15. doi: 10.1016/j.ohc.2004.08.004. PubMed PMID: 15763194.

262. Skarmoutsos F, Durnian JM, Damato BE. Delayed treatment of choroidal melanoma due to pregnancy. Eur J Ophthalmol. 2006;16(6):876-8. Epub 2006/12/28. PubMed PMID: 17191199.

263. Smith SL, Damato BE, Scholes AG, Nunn J, Field JK, Heighway J. Decreased endothelin receptor B expression in large primary uveal melanomas is associated with early clinical metastasis and short survival. Br J Cancer. 2002;87(11):1308-13. Epub 2002/11/20. doi: 10.1038/sj.bjc.6600620. PubMed PMID: 12439722; PubMed Central PMCID: PMC2408898.

264. Wright PK, Damato BE. Auditing outcomes after treatment of Scottish patients with uveal melanoma in Liverpool. J R Coll Surg Edinb. 1999;44(4):260-4. Epub 1999/08/24. PubMed PMID: 10453150.

265. Yang YC, Kent D, Fenerty CH, Kosmin AS, Damato BE. Pulsatile ocular blood flow in eyes with untreated choroidal melanoma. Eye (Lond). 1997;11 ( Pt 3):331-4. Epub 1997/01/01. doi: 10.1038/eye.1997.70. PubMed PMID: 9373471.

266. Ajani UA, Seddon JM, Hsieh CC, Egan KM, Albert DM, Gragoudas ES. Occupation and risk of uveal melanoma. An exploratory study. Cancer. 1992;70(12):2891-900. Epub 1992/12/15. PubMed PMID: 1451071.

267. Char DH, Gragoudas ES, Phillips TL. Uveal melanoma management. Br J Ophthalmol. 1989;73(6):476-7. Epub 1989/06/01. PubMed PMID: 2751984; PubMed Central PMCID: PMC1041773.

268. Chess J, Henkind P, Albert DM, Gragoudas ES, Reidel K, Weiss J, et al. Uveal melanoma presenting after cataract extraction with intraocular lens implantation. Ophthalmology. 1985;92(6):827-30. Epub 1985/06/01. PubMed PMID: 3897937.

269. Egan KM, Gragoudas ES, Seddon JM, Glynn RJ, Munzenreider JE, Goitein M, et al. The risk of enucleation after proton beam irradiation of uveal melanoma. Ophthalmology. 1989;96(9):1377-82; discussion 82-3. Epub 1989/09/01. PubMed PMID: 2550868.

270. Egan KM, Gragoudas ES, Seddon JM, Walsh SM. Smoking and the risk of early metastases from uveal melanoma. Ophthalmology. 1992;99(4):537-41. Epub 1992/04/01. PubMed PMID: 1584571.

271. Egan KM, Quinn JL, Gragoudas ES. Childbearing history associated with improved survival in choroidal melanoma. Arch Ophthalmol. 1999;117(7):939-42. Epub 1999/07/17. PubMed PMID: 10408460.

272. Egan KM, Ryan LM, Gragoudas ES. Survival implications of enucleation after definitive radiotherapy for choroidal melanoma: an example of regression on time-dependent covariates. Arch Ophthalmol. 1998;116(3):366-70. Epub 1998/03/26. PubMed PMID: 9514491.

273. Egan KM, Seddon JM, Glynn RJ, Gragoudas ES, Albert DM. Epidemiologic aspects of uveal melanoma. Surv Ophthalmol. 1988;32(4):239-51. Epub 1988/01/01. PubMed PMID: 3279559.

274. Egan KM, Walsh SM, Seddon JM, Gragoudas ES. An evaluation of the influence of reproductive factors on the risk of metastases from uveal melanoma. Ophthalmology. 1993;100(8):1160-5; discussion 6. Epub 1993/08/01. PubMed PMID: 8341495.

275. Foster BS, Gragoudas ES, Young LH. Photodynamic therapy of choroidal melanoma. Int Ophthalmol Clin. 1997;37(4):117-26. Epub 1998/02/28. PubMed PMID: 9480300.

276. Glynn RJ, Seddon JM, Gragoudas ES, Egan KM, Hart LJ. Evaluation of tumor regression and other prognostic factors for early and late metastasis after proton irradiation of uveal melanoma. Ophthalmology. 1989;96(10):1566-73. Epub 1989/10/01. PubMed PMID: 2685710.

277. Gragoudas ES. The Bragg peak of proton beams for treatment of uveal melanoma. Int Ophthalmol Clin. 1980;20(2):123-33. Epub 1980/01/01. PubMed PMID: 6249770.

278. Gragoudas ES. A randomized, controlled trial of varying radiation doses in the treatment of choroidal melanoma. Trans Am Ophthalmol Soc. 1998;96:691-720. Epub 1999/06/09. PubMed PMID: 10360306; PubMed Central PMCID: PMC1298412.

279. Gragoudas ES, Egan KM. Uveal melanoma: a rare malignancy. Ophthalmology. 2000;107(8):1441-2. Epub 2000/08/02. PubMed PMID: 10919884.

280. Gragoudas ES, Egan KM, Arrigg PG, Seddon JM, Glynn RJ, Munzenrider JE. Cataract extraction after proton beam irradiation for malignant melanoma of the eye. Arch Ophthalmol. 1992;110(4):475-9. Epub 1992/04/01. PubMed PMID: 1562251.

281. Gragoudas ES, Egan KM, Saornil MA, Walsh SM, Albert DM, Seddon JM. The time course of irradiation changes in proton beam-treated uveal melanomas. Ophthalmology. 1993;100(10):1555-9; discussion 60. Epub 1993/10/01. PubMed PMID: 8414415.

282. Gragoudas ES, Egan KM, Seddon JM, Glynn RJ, Walsh SM, Finn SM, et al. Survival of patients with metastases from uveal melanoma. Ophthalmology. 1991;98(3):383-9; discussion 90. Epub 1991/03/01. PubMed PMID: 2023760.

283. Gragoudas ES, Egan KM, Seddon JM, Walsh SM, Munzenrider JE. Intraocular recurrence of uveal melanoma after proton beam irradiation. Ophthalmology. 1992;99(5):760-6. Epub 1992/05/01. PubMed PMID: 1594223.

284. Gragoudas ES, Egan KM, Walsh SM, Regan S, Munzenrider JE, Taratuta V. Lens changes after proton beam irradiation for uveal melanoma. Am J Ophthalmol. 1995;119(2):157-64. Epub 1995/02/01. PubMed PMID: 7832221.

285. Gragoudas ES, Lane AM, Regan S, Li W, Judge HE, Munzenrider JE, et al. A randomized controlled trial of varying radiation doses in the treatment of choroidal melanoma. Arch Ophthalmol. 2000;118(6):773-8. Epub 2000/06/24. PubMed PMID: 10865313.

286. Gragoudas ES, Li W, Lane AM, Munzenrider J, Egan KM. Risk factors for radiation maculopathy and papillopathy after intraocular irradiation. Ophthalmology. 1999;106(8):1571-7; discussion 7-8. Epub 1999/08/12. doi: 10.1016/s0161-6420(99)90455-4. PubMed PMID: 10442906.

287. Gragoudas ES, Marie Lane A. Uveal melanoma: proton beam irradiation. Ophthalmol Clin North Am. 2005;18(1):111-8, ix. Epub 2005/03/15. doi: 10.1016/j.ohc.2004.08.002. PubMed PMID: 15763196.

288. Gragoudas ES, Seddon JM, Egan KM, Glynn RJ, Goitein M, Munzenrider J, et al. Metastasis from uveal melanoma after proton beam irradiation. Ophthalmology. 1988;95(7):992-9. Epub 1988/07/01. PubMed PMID: 2845324.

289. Guyer DR, Mukai S, Egan KM, Seddon JM, Walsh SM, Gragoudas ES. Radiation maculopathy after proton beam irradiation for choroidal melanoma. Ophthalmology. 1992;99(8):1278-85. Epub 1992/08/01. PubMed PMID: 1325044.

290. Haimovici R, Mukai S, Schachat AP, Haynie GD, Thomas MA, Meredith TA, et al. Rhegmatogenous retinal detachment in eyes with uveal melanoma. Retina. 1996;16(6):488-96. Epub 1996/01/01. PubMed PMID: 9002131.

291. Haynie GD, Shen TT, Gragoudas ES, Young LH. Flow cytometry analysis of peripheral blood lymphocytes in patients with choroidal melanoma. Am J Ophthalmol. 1997;124(3):357-61. Epub 1998/01/24. PubMed PMID: 9439361.

292. Hu L, Wu X, Song Y, Young LH, Gragoudas ES. [Photodynamic therapy of pigmented choroidal melanomas in rabbits]. Zhonghua Yan Ke Za Zhi. 2002;38(8):491-4. Epub 2002/11/02. PubMed PMID: 12410990.

293. Hu LK, Hasan T, Gragoudas ES, Young LH. Photoimmunotherapy of human uveal melanoma cells. Exp Eye Res. 1995;61(4):385-91. Epub 1995/10/01. PubMed PMID: 8549679.

294. Hu LK, Huh K, Gragoudas ES, Young LH. Establishment of pigmented choroidal melanomas in a rabbit model. Retina. 1994;14(3):264-9. Epub 1994/01/01. PubMed PMID: 7973123.

295. Kim IK, Lane AM, Egan KM, Munzenrider J, Gragoudas ES. Natural history of radiation papillopathy after proton beam irradiation of parapapillary melanoma. Ophthalmology. 2010;117(8):1617-22. Epub 2010/04/07. doi: 10.1016/j.ophtha.2009.12.015. PubMed PMID: 20363507.

296. Kim IK, Lane AM, Gragoudas ES. Survival in patients with presymptomatic diagnosis of metastatic uveal melanoma. Arch Ophthalmol. 2010;128(7):871-5. Epub 2010/07/14. doi: 10.1001/archophthalmol.2010.121. PubMed PMID: 20625048.

297. Kolodny NH, Gragoudas ES, D'Amico DJ, Seddon JM, Minichiello M, Murphy EJ, et al. Preliminary results on phosphorus-31 nuclear magnetic resonance evaluation of human uveal melanoma in enucleated eyes. Ophthalmology. 1988;95(5):666-73. Epub 1988/05/01. PubMed PMID: 3174026.

298. Krause M, Kwong KK, Xiong J, Gragoudas ES, Young LH. MRI of blood volume and cellular uptake of superparamagnetic iron in an animal model of choroidal melanoma. Ophthalmic Res. 2002;34(4):241-50. Epub 2002/09/26. doi: 63883. PubMed PMID: 12297697.

299. Krause MH, Kwong KK, Gragoudas ES, Young LH. MRI of blood volume with superparamagnetic iron in choroidal melanoma treated with thermotherapy. Magn Reson Imaging. 2004;22(6):779-87. Epub 2004/07/06. doi: 10.1016/j.mri.2004.01.052. PubMed PMID: 15234446.

300. Krause MH, Kwong KK, Xiong J, Gragoudas ES, Young LH. MRI of blood volume with MS 325 in experimental choroidal melanoma. Magn Reson Imaging. 2003;21(7):725-32. Epub 2003/10/16. PubMed PMID: 14559336.

301. Krause MH, Xiong J, Gragoudas ES, Young LH. Treatment of experimental choroidal melanoma with an Nd:yttrium-lanthanum-fluoride laser at 1047 nm. Arch Ophthalmol. 2003;121(3):357-63. Epub 2003/03/06. PubMed PMID: 12617706.

302. Lane AM, Egan KM, Harmon D, Holbrook A, Munzenrider JE, Gragoudas ES. Adjuvant interferon therapy for patients with uveal melanoma at high risk of metastasis. Ophthalmology. 2009;116(11):2206-12. Epub 2009/09/12. doi: 10.1016/j.ophtha.2009.04.044. PubMed PMID: 19744725.

303. Lane AM, Egan KM, Kim IK, Gragoudas ES. Mortality after diagnosis of small melanocytic lesions of the choroid. Arch Ophthalmol. 2010;128(8):996-1000. Epub 2010/08/11. doi: 10.1001/archophthalmol.2010.166. PubMed PMID: 20696999.

304. Lane AM, Egan KM, Yang J, Saornil MA, Alroy J, Albert D, et al. An evaluation of tumour vascularity as a prognostic indicator in uveal melanoma. Melanoma Res. 1997;7(3):237-42. Epub 1997/06/01. PubMed PMID: 9195563.

305. Levin LA, Gragoudas ES, Lessell S. Endothelial cell loss in irradiated optic nerves. Ophthalmology. 2000;107(2):370-4. Epub 2000/02/26. PubMed PMID: 10690841.

306. Li W, Gragoudas ES, Egan KM. Metastatic melanoma death rates by anatomic site after proton beam irradiation for uveal melanoma. Arch Ophthalmol. 2000;118(8):1066-70. Epub 2000/08/02. PubMed PMID: 10922199.

307. Li W, Gragoudas ES, Egan KM. Tumor basal area and metastatic death after proton beam irradiation for choroidal melanoma. Arch Ophthalmol. 2003;121(1):68-72. Epub 2003/01/14. PubMed PMID: 12523887.

308. Li W, Judge H, Gragoudas ES, Seddon JM, Egan KM. Patterns of tumor initiation in choroidal melanoma. Cancer Res. 2000;60(14):3757-60. Epub 2000/08/05. PubMed PMID: 10919647.

309. Lischko AM, Seddon JM, Gragoudas ES, Egan KM, Glynn RJ. Evaluation of prior primary malignancy as a determinant of uveal melanoma. A case-control study. Ophthalmology. 1989;96(12):1716-21. Epub 1989/12/01. PubMed PMID: 2622616.

310. Lubin JR, Gragoudas ES, Albert DM. Choroidal neovascularization associated with malignant melanoma: a case report. Acta Ophthalmol (Copenh). 1982;60(3):412-8. Epub 1982/06/01. PubMed PMID: 6182734.

311. Marucci L, Ancukiewicz M, Lane AM, Collier JM, Gragoudas ES, Munzenrider JE. Uveal melanoma recurrence after fractionated proton beam therapy: comparison of survival in patients treated with reirradiation or with enucleation. Int J Radiat Oncol Biol Phys. 2011;79(3):842-6. Epub 2010/05/18. doi: 10.1016/j.ijrobp.2009.12.018. PubMed PMID: 20472356.

312. Marucci L, Lane AM, Li W, Egan KM, Gragoudas ES, Adams JA, et al. Conservation treatment of the eye: Conformal proton reirradiation for recurrent uveal melanoma. Int J Radiat Oncol Biol Phys. 2006;64(4):1018-22. Epub 2005/12/27. doi: 10.1016/j.ijrobp.2005.09.035. PubMed PMID: 16376492.

313. Morgan CM, Gragoudas ES. Limited choroidal hemorrhage mistaken for a choroidal melanoma. Ophthalmology. 1987;94(1):41-6. Epub 1987/01/01. PubMed PMID: 3550566.

314. Munzenrider JE, Gragoudas ES, Seddon JM, Sisterson J, McNulty P, Birnbaum S, et al. Conservative treatment of uveal melanoma: probability of eye retention after proton treatment. Int J Radiat Oncol Biol Phys. 1988;15(3):553-8. Epub 1988/09/01. PubMed PMID: 2843486.

315. Munzenrider JE, Verhey LJ, Gragoudas ES, Seddon JM, Urie M, Gentry R, et al. Conservative treatment of uveal melanoma: local recurrence after proton beam therapy. Int J Radiat Oncol Biol Phys. 1989;17(3):493-8. Epub 1989/09/01. PubMed PMID: 2550395.

316. Panagopoulos JA, Svitra PP, Puliafito CA, Gragoudas ES. Photodynamic therapy for experimental intraocular melanoma using chloroaluminum sulfonated phthalocyanine. Arch Ophthalmol. 1989;107(6):886-90. Epub 1989/06/01. PubMed PMID: 2730407.

317. Park SS, Theodossiadis PG, Gragoudas ES. Intrascleral foreign body simulating extrascleral extension of uveal melanoma. Arch Ophthalmol. 1994;112(12):1620-1. Epub 1994/12/01. PubMed PMID: 7993220.

318. Park SS, Walsh SM, Gragoudas ES. Visual-field deficits associated with proton beam irradiation for parapapillary choroidal melanoma. Ophthalmology. 1996;103(1):110-6. Epub 1996/01/01. PubMed PMID: 8628541.

319. Pineda R, 2nd, Theodossiadis PG, Gonzalez VH, Hu LK, Hart LJ, Gragoudas ES, et al. Establishment of a rabbit model of extrascleral extension of ocular melanoma. Retina. 1998;18(4):368-72. Epub 1998/09/08. PubMed PMID: 9730182.

320. Polivogianis L, Seddon JM, Glynn RJ, Gragoudas ES, Albert DM. Comparison of transillumination and histologic slide measurements of tumor diameter in uveal melanoma. Ophthalmology. 1988;95(11):1576-82. Epub 1988/11/01. PubMed PMID: 3062526.

321. Regan S, Judge HE, Gragoudas ES, Egan KM. Iris color as a prognostic factor in ocular melanoma. Arch Ophthalmol. 1999;117(6):811-4. Epub 1999/06/16. PubMed PMID: 10369595.

322. Reinke MH, Gragoudas ES. Unusual hemorrhagic lesions masquerading as choroidal melanoma. Int Ophthalmol Clin. 1997;37(4):135-47. Epub 1998/01/16. PubMed PMID: 9429937.

323. Riedel KG, Svitra PP, Seddon JM, Albert DM, Gragoudas ES, Koehler AM, et al. Proton beam irradiation and hyperthermia. Effects on experimental choroidal melanoma. Arch Ophthalmol. 1985;103(12):1862-9. Epub 1985/12/01. PubMed PMID: 3000327.

324. Saornil MA, Egan KM, Gragoudas ES, Seddon JM, Walsh SM, Albert DM. Histopathology of proton beam-irradiated vs enucleated uveal melanomas. Arch Ophthalmol. 1992;110(8):1112-8. Epub 1992/08/01. PubMed PMID: 1323252.

325. Schmidt-Erfurth U, Bauman W, Gragoudas E, Flotte TJ, Michaud NA, Birngruber R, et al. Photodynamic therapy of experimental choroidal melanoma using lipoprotein-delivered benzoporphyrin. Ophthalmology. 1994;101(1):89-99. Epub 1994/01/01. PubMed PMID: 8302569.

326. Seddon J, Gragoudas E, Egan K, Polivogianis L, Finn S, Albert D. Standardized data collection and coding in eye disease epidemiology: the Uveal Melanoma Data System. Ophthalmic Surg. 1991;22(3):127-36. Epub 1991/03/01. PubMed PMID: 2030892.

327. Seddon JM, Gragoudas E, Albert D, Hsieh CC, Polivogianis L, Egan K. Echographic and histologic tumor height measurements in uveal melanoma. Am J Ophthalmol. 1986;101(1):126-8. Epub 1986/01/15. PubMed PMID: 3942170.

328. Seddon JM, Gragoudas ES, Albert DM. Ciliary body and choroidal melanomas treated by proton beam irradiation. Histopathologic study of eyes. Arch Ophthalmol. 1983;101(9):1402-8. Epub 1983/09/01. PubMed PMID: 6311146.

329. Seddon JM, Gragoudas ES, Albert DM, Hsieh CC, Polivogianis L, Friedenberg GR. Comparison of survival rates for patients with uveal melanoma after treatment with proton beam irradiation or enucleation. Am J Ophthalmol. 1985;99(3):282-90. Epub 1985/03/15. PubMed PMID: 2983558.

330. Seddon JM, Gragoudas ES, Egan KM, Glynn RJ, Howard S, Fante RG, et al. Relative survival rates after alternative therapies for uveal melanoma. Ophthalmology. 1990;97(6):769-77. Epub 1990/06/01. PubMed PMID: 2374681.

331. Seddon JM, Gragoudas ES, Glynn RJ, Egan KM, Albert DM, Blitzer PH. Host factors, UV radiation, and risk of uveal melanoma. A case-control study. Arch Ophthalmol. 1990;108(9):1274-80. Epub 1990/09/01. PubMed PMID: 2400347.

332. Seddon JM, Gragoudas ES, Polivogianis L, Hsieh CC, Egan KM, Goitein M, et al. Visual outcome after proton beam irradiation of uveal melanoma. Ophthalmology. 1986;93(5):666-74. Epub 1986/05/01. PubMed PMID: 3014415.

333. Seddon JM, MacLaughlin DT, Albert DM, Gragoudas ES, Ference M, 3rd. Uveal melanomas presenting during pregnancy and the investigation of oestrogen receptors in melanomas. Br J Ophthalmol. 1982;66(11):695-704. Epub 1982/11/01. PubMed PMID: 7126514; PubMed Central PMCID: PMC1039903.

334. Seddon JM, Polivogianis L, Hsieh CC, Albert DM, Gamel JW, Gragoudas ES. Death from uveal melanoma. Number of epithelioid cells and inverse SD of nucleolar area as prognostic factors. Arch Ophthalmol. 1987;105(6):801-6. Epub 1987/06/01. PubMed PMID: 3579712.

335. Smith EV, Kolodny NH, Gragoudas ES, Egan KM, Finn S, D'Amico DJ, et al. An NMR blood test for uveal melanoma? Invest Ophthalmol Vis Sci. 1990;31(5):993-7. Epub 1990/05/01. PubMed PMID: 2335461.

336. Smith EV, Kolodny NH, Gragoudas ES, Rubin LG, Seddon JH, D'Amico DJ. Safety and effectiveness of magnetic resonance imaging of choroidal melanoma patients with episcleral tantalum rings after proton beam irradiation. Am J Ophthalmol. 1988;105(6):695-6. Epub 1988/06/15. PubMed PMID: 2837092.

337. Suit H, Goitein M, Munzenrider J, Verhey L, Blitzer P, Gragoudas E, et al. Evaluation of the clinical applicability of proton beams in definitive fractionated radiation therapy. Int J Radiat Oncol Biol Phys. 1982;8(12):2199-205. Epub 1982/12/01. PubMed PMID: 6298160.

338. Suit HD, Goitein M, Munzenrider J, Verhey L, Urie M, Gragoudas E, et al. Increased efficacy of radiation therapy by use of proton beam. Strahlenther Onkol. 1990;166(1):40-4. Epub 1990/01/01. PubMed PMID: 2154047.

339. Vavvas D, Kim I, Lane AM, Chaglassian A, Mukai S, Gragoudas E. Posterior uveal melanoma in young patients treated with proton beam therapy. Retina. 2010;30(8):1267-71. Epub 2010/03/13. doi: 10.1097/IAE.0b013e3181cfdfad. PubMed PMID: 20224468.

340. Wang X, Egan KM, Gragoudas ES, Kelsey KT. Constitutional alterations in p16 in patients with uveal melanoma. Melanoma Res. 1996;6(6):405-10. Epub 1996/12/01. PubMed PMID: 9013477.

341. Weissgold DJ, Gragoudas ES, Green JP, Kent CJ, Rubin PA. Eye-sparing treatment of massive extrascleral extension of choroidal melanoma. Arch Ophthalmol. 1998;116(4):531-3. Epub 1998/05/02. PubMed PMID: 9565057.

342. Young LH, Egan KM, Walsh SM, Gragoudas ES. Familial uveal melanoma. Am J Ophthalmol. 1994;117(4):516-20. Epub 1994/04/15. PubMed PMID: 8154536.

343. Young LH, Gragoudas ES. Macular uveal melanoma treated with proton beam irradiation. 10-year follow-up observation with histopathologic correlation. Retina. 1994;14(1):43-6. Epub 1994/01/01. PubMed PMID: 8016461.

344. Young LH, Howard MA, Hu LK, Kim RY, Gragoudas ES. Photodynamic therapy of pigmented choroidal melanomas using a liposomal preparation of benzoporphyrin derivative. Arch Ophthalmol. 1996;114(2):186-92. Epub 1996/02/01. PubMed PMID: 8573023.

345. Zacks DN, Pinnolis MK, Berson EL, Gragoudas ES. Melanoma-associated retinopathy and recurrent exudative retinal detachments in a patient with choroidal melanoma. Am J Ophthalmol. 2001;132(4):578-81. Epub 2001/10/09. PubMed PMID: 11589887.

346. Accuracy of diagnosis of choroidal melanomas in the Collaborative Ocular Melanoma Study. COMS report no. 1. Arch Ophthalmol. 1990;108(9):1268-73. Epub 1990/09/01. PubMed PMID: 2205183.

347. Design and methods of a clinical trial for a rare condition: the Collaborative Ocular Melanoma Study. COMS Report No. 3. Control Clin Trials. 1993;14(5):362-91. Epub 1993/10/01. PubMed PMID: 8222668.

348. Factors predictive of growth and treatment of small choroidal melanoma: COMS Report No. 5. The Collaborative Ocular Melanoma Study Group. Arch Ophthalmol. 1997;115(12):1537-44. Epub 1997/12/24. PubMed PMID: 9400787.

349. Mortality in patients with small choroidal melanoma. COMS report no. 4. The Collaborative Ocular Melanoma Study Group. Arch Ophthalmol. 1997;115(7):886-93. Epub 1997/07/01. PubMed PMID: 9230829.

350. The Collaborative Ocular Melanoma Study (COMS) randomized trial of pre-enucleation radiation of large choroidal melanoma III: local complications and observations following enucleation COMS report no. 11. Am J Ophthalmol. 1998;126(3):362-72. Epub 1998/09/23. PubMed PMID: 9744369.

351. The Collaborative Ocular Melanoma Study (COMS) randomized trial of pre-enucleation radiation of large choroidal melanoma II: initial mortality findings. COMS report no. 10. Am J Ophthalmol. 1998;125(6):779-96. Epub 1998/06/30. PubMed PMID: 9645716.

352. The Collaborative Ocular Melanoma Study (COMS) randomized trial of pre-enucleation radiation of large choroidal melanoma I: characteristics of patients enrolled and not enrolled. COMS report no. 9. Am J Ophthalmol. 1998;125(6):767-78. Epub 1998/06/30. PubMed PMID: 9645715.

353. Histopathologic characteristics of uveal melanomas in eyes enucleated from the Collaborative Ocular Melanoma Study. COMS report no. 6. Am J Ophthalmol. 1998;125(6):745-66. Epub 1998/06/30. PubMed PMID: 9645714.

354. Echography (Ultrasound) Procedures for the Collaborative Ocular Melanoma Study (COMS), Report no. 12, Part II. J Ophthalmic Nurs Technol. 1999;18(5):219-32. Epub 2000/06/10. PubMed PMID: 10847049.

355. Echography (ultrasound) procedures for the Collaborative Ocular Melanoma Study (COMS), Report no. 12, Part I. J Ophthalmic Nurs Technol. 1999;18(4):143-9. Epub 2000/06/10. PubMed PMID: 10847038.

356. Quality of life assessment in the collaborative ocular melanoma study: design and methods. COMS-QOLS Report No. 1. COMS Quality of Life Study Group. Ophthalmic Epidemiol. 1999;6(1):5-17. Epub 1999/06/29. PubMed PMID: 10384680.

357. Sociodemographic and clinical predictors of participation in two randomized trials: findings from the Collaborative Ocular Melanoma Study COMS report no. 7. Control Clin Trials. 2001;22(5):526-37. Epub 2001/10/02. PubMed PMID: 11578786.

358. Assessment of metastatic disease status at death in 435 patients with large choroidal melanoma in the Collaborative Ocular Melanoma Study (COMS): COMS report no. 15. Arch Ophthalmol. 2001;119(5):670-6. Epub 2001/05/18. PubMed PMID: 11346394.

359. Comparison of clinical, echographic, and histopathological measurements from eyes with medium-sized choroidal melanoma in the collaborative ocular melanoma study: COMS report no. 21. Arch Ophthalmol. 2003;121(8):1163-71. Epub 2003/08/13. doi: 10.1001/archopht.121.8.1163. PubMed PMID: 12912695.

360. Trends in size and treatment of recently diagnosed choroidal melanoma, 1987-1997: findings from patients examined at collaborative ocular melanoma study (COMS) centers: COMS report no. 20. Arch Ophthalmol. 2003;121(8):1156-62. Epub 2003/08/13. doi: 10.1001/archopht.121.8.1156. PubMed PMID: 12912694.

361. Ten-year follow-up of fellow eyes of patients enrolled in Collaborative Ocular Melanoma Study randomized trials: COMS report no. 22. Ophthalmology. 2004;111(5):966-76. Epub 2004/05/04. doi: 10.1016/j.ophtha.2003.08.029. PubMed PMID: 15121376.

362. The COMS randomized trial of iodine 125 brachytherapy for choroidal melanoma: V. Twelve-year mortality rates and prognostic factors: COMS report No. 28. Arch Ophthalmol. 2006;124(12):1684-93. Epub 2006/12/13. doi: 10.1001/archopht.124.12.1684. PubMed PMID: 17159027.

363. Incidence of cataract and outcomes after cataract surgery in the first 5 years after iodine 125 brachytherapy in the Collaborative Ocular Melanoma Study: COMS Report No. 27. Ophthalmology. 2007;114(7):1363-71. Epub 2007/03/06. doi: 10.1016/j.ophtha.2006.10.039. PubMed PMID: 17337065.

364. Ajluni PB. Nonprofit and for-profit COMs: investing in the future of osteopathic medicine. J Am Osteopath Assoc. 2007;107(10):425-6. Epub 2007/10/25. PubMed PMID: 17956993.

365. Astrahan MA. Improved treatment planning for COMS eye plaques. Int J Radiat Oncol Biol Phys. 2005;61(4):1227-42. Epub 2005/03/09. doi: 10.1016/j.ijrobp.2004.09.062. PubMed PMID: 15752905.

366. Astrahan MA, Szechter A, Finger PT. Design and dosimetric considerations of a modified COMS plaque: the reusable "seed-guide" insert. Med Phys. 2005;32(8):2706-16. Epub 2005/10/01. PubMed PMID: 16193802.

367. Avery RB, Diener-West M, Reynolds SM, Grossniklaus HE, Green WR, Albert DM. Histopathologic characteristics of choroidal melanoma in eyes enucleated after iodine 125 brachytherapy in the collaborative ocular melanoma study. Arch Ophthalmol. 2008;126(2):207-12. Epub 2008/02/13. doi: 10.1001/archophthalmol.2007.50. PubMed PMID: 18268211.

368. Baldwin L. COMS Junior Research Associates Program. J Osteopath (Kirksvill). 1962;69:50-3. Epub 1962/11/01. PubMed PMID: 13969337.

369. Beiki-Ardakani A, Jezioranski J, Jaffray DA, Young I. Improving quality assurance for assembled COMS eye plaques using a pinhole gamma camera. Med Phys. 2008;35(10):4318-23. Epub 2008/11/04. PubMed PMID: 18975677.

370. Bell H. The docs behind the dot-coms. Hosp Pract (1995). 2000;35(3):17-24. Epub 2000/03/29. PubMed PMID: 10737245.

371. Benson WE. Is the Collaborative Ocular Melanoma Study worthwhile? Arch Ophthalmol. 1986;104(12):1745-7. Epub 1986/12/01. PubMed PMID: 3789966.

372. Benson WE. The COMS: why was it not stopped sooner? Arch Ophthalmol. 2002;120(5):672-3; author reply 3. Epub 2002/05/11. PubMed PMID: 12003630.

373. Boldt HC, Byrne SF, Gilson MM, Finger PT, Green RL, Straatsma BR, et al. Baseline echographic characteristics of tumors in eyes of patients enrolled in the Collaborative Ocular Melanoma Study: COMS report no. 29. Ophthalmology. 2008;115(8):1390-7, 7 e1-2. Epub 2008/02/13. doi: 10.1016/j.ophtha.2007.12.015. PubMed PMID: 18267342.

374. Boldt HC, Melia BM, Liu JC, Reynolds SM. I-125 brachytherapy for choroidal melanoma photographic and angiographic abnormalities: the Collaborative Ocular Melanoma Study: COMS Report No. 30. Ophthalmology. 2009;116(1):106-15 e1. Epub 2009/01/03. doi: 10.1016/j.ophtha.2008.10.013. PubMed PMID: 19118701; PubMed Central PMCID: PMC3202984.

375. Byrne SF, Marsh MJ, Boldt HC, Green RL, Johnson RN, Wilson DJ. Consistency of observations from echograms made centrally in the Collaborative Ocular Melanoma Study COMS Report No. 13. Ophthalmic Epidemiol. 2002;9(1):11-27. Epub 2002/01/30. PubMed PMID: 11815892.

376. Damato B. Legacy of the collaborative ocular melanoma study. Arch Ophthalmol. 2007;125(7):966-8. Epub 2007/07/11. doi: 10.1001/archopht.125.7.966. PubMed PMID: 17620581.

377. Davis MD, Fine SL, Kupfer C. The collaborative Ocular Melanoma Study. Arch Ophthalmol. 1994;112(6):730-1. Epub 1994/06/01. PubMed PMID: 8002824.

378. Diener-West M, Connor PB, Newhouse MM, Hawkins BS. Feasibility of keying data from screen-displayed facsimile images in an ongoing trial: the collaborative ocular melanoma study. Control Clin Trials. 1998;19(1):39-49. Epub 1998/03/11. PubMed PMID: 9492968.

379. Diener-West M, Earle JD, Fine SL, Hawkins BS, Moy CS, Reynolds SM, et al. The COMS randomized trial of iodine 125 brachytherapy for choroidal melanoma, III: initial mortality findings. COMS Report No. 18. Arch Ophthalmol. 2001;119(7):969-82. Epub 2001/07/27. PubMed PMID: 11448319.

380. Diener-West M, Earle JD, Fine SL, Hawkins BS, Moy CS, Reynolds SM, et al. The COMS randomized trial of iodine 125 brachytherapy for choroidal melanoma, II: characteristics of patients enrolled and not enrolled. COMS Report No. 17. Arch Ophthalmol. 2001;119(7):951-65. Epub 2001/07/27. PubMed PMID: 11448318.

381. Diener-West M, Reynolds SM, Agugliaro DJ, Caldwell R, Cumming K, Earle JD, et al. Screening for metastasis from choroidal melanoma: the Collaborative Ocular Melanoma Study Group Report 23. J Clin Oncol. 2004;22(12):2438-44. Epub 2004/06/16. doi: 10.1200/jco.2004.08.194. PubMed PMID: 15197206.

382. Diener-West M, Reynolds SM, Agugliaro DJ, Caldwell R, Cumming K, Earle JD, et al. Development of metastatic disease after enrollment in the COMS trials for treatment of choroidal melanoma: Collaborative Ocular Melanoma Study Group Report No. 26. Arch Ophthalmol. 2005;123(12):1639-43. Epub 2005/12/14. doi: 10.1001/archopht.123.12.1639. PubMed PMID: 16344433.

383. Diener-West M, Reynolds SM, Agugliaro DJ, Caldwell R, Cumming K, Earle JD, et al. Second primary cancers after enrollment in the COMS trials for treatment of choroidal melanoma: COMS Report No. 25. Arch Ophthalmol. 2005;123(5):601-4. Epub 2005/05/11. doi: 10.1001/archopht.123.5.601. PubMed PMID: 15883277.

384. D'Souza C, Nakano MM, Frisby DL, Zuber P. Translation of the open reading frame encoded by comS, a gene of the srf operon, is necessary for the development of genetic competence, but not surfactin biosynthesis, in Bacillus subtilis. J Bacteriol. 1995;177(14):4144-8. Epub 1995/07/01. PubMed PMID: 7608091; PubMed Central PMCID: PMC177150.

385. D'Souza C, Nakano MM, Zuber P. Identification of comS, a gene of the srfA operon that regulates the establishment of genetic competence in Bacillus subtilis. Proc Natl Acad Sci U S A. 1994;91(20):9397-401. Epub 1994/09/27. PubMed PMID: 7937777; PubMed Central PMCID: PMC44819.

386. Earle J, Kline RW, Robertson DM. Selection of iodine 125 for the Collaborative Ocular Melanoma Study. Arch Ophthalmol. 1987;105(6):763-4. Epub 1987/06/01. PubMed PMID: 3579705.

387. Earle JD. Results from the Collaborative Ocular Melanoma Study (COMS) of enucleation versus preoperative radiation therapy in the management of large ocular melanomas. Int J Radiat Oncol Biol Phys. 1999;43(5):1168-9. Epub 1999/04/07. PubMed PMID: 10192370.

388. Fine SL. Collaborative ocular melanoma study group. Arch Fam Med. 1999;8(1):11-2. Epub 1999/02/05. PubMed PMID: 9932065.

389. Fine SL, Hawkins BS. The investigators' perspective on the collaborative ocular melanoma study. Arch Ophthalmol. 2007;125(7):968-71. Epub 2007/07/11. doi: 10.1001/archopht.125.7.968. PubMed PMID: 17620582.

390. Fine SL, Straatsma BR, Earle JD, Hawkins BS, McLaughlin JA. Failure of preenucleation radiation to decrease uveal melanoma mortality. The Collaborative Ocular melanoma Study Steering Committee. Am J Ophthalmol. 1989;107(4):440-2. Epub 1989/04/15. PubMed PMID: 2929718.

391. Gilson MM, Diener-West M, Hawkins BS. Comparison of survival among eligible patients not enrolled versus enrolled in the Collaborative Ocular Melanoma Study (COMS) randomized trial of pre-enucleation radiation of large choroidal melanoma. Ophthalmic Epidemiol. 2007;14(4):251-7. Epub 2007/09/27. doi: 10.1080/01658100701473275. PubMed PMID: 17896305.

392. Grossniklaus HE, Albert DM, Green WR, Conway BP, Hovland KR. Clear cell differentiation in choroidal melanoma. COMS report no. 8. Collaborative Ocular Melanoma Study Group. Arch Ophthalmol. 1997;115(7):894-8. Epub 1997/07/01. PubMed PMID: 9230830.

393. Grostern RJ, Slusker Shternfeld I, Bacus SS, Gilchrist K, Zimbric ML, Albert DM. Absence of type I estrogen receptors in choroidal melanoma: analysis of Collaborative Ocular Melanoma Study (COMS) eyes. Am J Ophthalmol. 2001;131(6):788-91. Epub 2001/06/01. PubMed PMID: 11384577.

394. Hamoen LW, Eshuis H, Jongbloed J, Venema G, van Sinderen D. A small gene, designated comS, located within the coding region of the fourth amino acid-activation domain of srfA, is required for competence development in Bacillus subtilis. Mol Microbiol. 1995;15(1):55-63. Epub 1995/01/01. PubMed PMID: 7752896.

395. Hawkins BS. The Collaborative Ocular Melanoma Study (COMS) randomized trial of pre-enucleation radiation of large choroidal melanoma: IV. Ten-year mortality findings and prognostic factors. COMS report number 24. Am J Ophthalmol. 2004;138(6):936-51. Epub 2005/01/05. doi: 10.1016/j.ajo.2004.07.006. PubMed PMID: 15629284.

396. Hawkins BS. Collaborative ocular melanoma study randomized trial of I-125 brachytherapy. Clin Trials. 2011;8(5):661-73. Epub 2011/10/21. doi: 10.1177/1740774511419684. PubMed PMID: 22013172.

397. Jampol LM, Moy CS, Murray TG, Reynolds SM, Albert DM, Schachat AP, et al. The COMS randomized trial of iodine 125 brachytherapy for choroidal melanoma: IV. Local treatment failure and enucleation in the first 5 years after brachytherapy. COMS report no. 19. Ophthalmology. 2002;109(12):2197-206. Epub 2002/12/06. PubMed PMID: 12466159.

398. Kleckner MS, Jr., Caddell DE, Billington CB, Fowler MW, Jr. The diagnostic and prognostic value of serial alterations of plasma cholinesterase and transaminases (SGO-T and SGP-T) in patients with hepatic coms. J Ky Med Assoc. 1962;60:861-3. Epub 1962/09/01. PubMed PMID: 14456694.

399. Krintz AL, Hanson WF, Ibbott GS, Followill DS. A reanalysis of the Collaborative Ocular Melanoma Study Medium Tumor Trial eye plaque dosimetry. Int J Radiat Oncol Biol Phys. 2003;56(3):889-98. Epub 2003/06/06. PubMed PMID: 12788199.

400. Lisowsky T. Pushing bodies through COMs. J Am Osteopath Assoc. 2008;108(3):105; discussion -6. Epub 2008/04/09. PubMed PMID: 18391080.

401. Liu J, Zuber P. A molecular switch controlling competence and motility: competence regulatory factors ComS, MecA, and ComK control sigmaD-dependent gene expression in Bacillus subtilis. J Bacteriol. 1998;180(16):4243-51. Epub 1998/08/08. PubMed PMID: 9696775; PubMed Central PMCID: PMC107423.

402. Liu L, Nakano MM, Lee OH, Zuber P. Plasmid-amplified comS enhances genetic competence and suppresses sinR in Bacillus subtilis. J Bacteriol. 1996;178(17):5144-52. Epub 1996/09/01. PubMed PMID: 8752331; PubMed Central PMCID: PMC178310.

403. Marcus DM, Minkovitz JB, Wardwell SD, Albert DM. The value of nucleolar organizer regions in uveal melanoma. The Collaborative Ocular Melanoma Study Group. Am J Ophthalmol. 1990;110(5):527-34. Epub 1990/11/15. PubMed PMID: 1700611.

404. Margo CE. The Collaborative Ocular Melanoma Study: an overview. Cancer Control. 2004;11(5):304-9. Epub 2004/09/21. PubMed PMID: 15377989.

405. Melhus CS, Rivard MJ. COMS eye plaque brachytherapy dosimetry simulations for 103Pd, 125I, and 131Cs. Med Phys. 2008;35(7):3364-71. Epub 2008/08/14. PubMed PMID: 18697560.

406. Melia BM, Abramson DH, Albert DM, Boldt HC, Earle JD, Hanson WF, et al. Collaborative ocular melanoma study (COMS) randomized trial of I-125 brachytherapy for medium choroidal melanoma. I. Visual acuity after 3 years COMS report no. 16. Ophthalmology. 2001;108(2):348-66. Epub 2001/02/13. PubMed PMID: 11158813.

407. Melia M, Moy CS, Reynolds SM, Cella D, Murray TG, Hovland KR, et al. Development and validation of disease-specific measures for choroidal melanoma: COMS-QOLS report No. 2. Arch Ophthalmol. 2003;121(7):1010-20. Epub 2003/07/16. doi: 10.1001/archopht.121.7.1010. PubMed PMID: 12860806.

408. Melia M, Moy CS, Reynolds SM, Hayman JA, Murray TG, Hovland KR, et al. Quality of life after iodine 125 brachytherapy vs enucleation for choroidal melanoma: 5-year results from the Collaborative Ocular Melanoma Study: COMS QOLS Report No. 3. Arch Ophthalmol. 2006;124(2):226-38. Epub 2006/02/16. doi: 10.1001/archopht.124.2.226. PubMed PMID: 16476893.

409. Menzies D. Was it really only a scant few years ago that Silicon Valley was booming, dot-coms were the next big things and Nortel shares were trading at more than $100? Hosp Q. 2003;6(3):81-4, 4. Epub 2003/07/09. PubMed PMID: 12846149.

410. Mobley RY, Moy CS, Reynolds SM, Diener-West M, Newhouse MM, Kerman JS, et al. Time trends in personnel certification and turnover in the Collaborative Ocular Melanoma Study. Clin Trials. 2004;1(4):377-86. Epub 2005/11/11. PubMed PMID: 16279276.

411. Mohr R, Meir O, Smolinsky A, Goor DA. A method for continuous on-line monitoring of systemic vascular resistance (COMS) after open heart procedures. J Cardiovasc Surg (Torino). 1987;28(5):558-65. Epub 1987/09/01. PubMed PMID: 3498725.

412. Moy CS, Albert DM, Diener-West M, McCaffrey LD, Scully RE, Willson JK. Cause-specific mortality coding. methods in the collaborative ocular melanoma study coms report no. 14. Control Clin Trials. 2001;22(3):248-62. Epub 2001/06/01. PubMed PMID: 11384789.

413. Ogura M, Liu L, Lacelle M, Nakano MM, Zuber P. Mutational analysis of ComS: evidence for the interaction of ComS and MecA in the regulation of competence development in Bacillus subtilis. Mol Microbiol. 1999;32(4):799-812. Epub 1999/06/11. PubMed PMID: 10361283.

414. Park WL. The Collaborative Ocular Melanoma Study. J Am Optom Assoc. 1992;63(9):609-10. Epub 1992/09/01. PubMed PMID: 1430749.

415. Payson SM. Marketing key to increased women applicants to COMs. J Am Osteopath Assoc. 1996;96(1):26. Epub 1996/01/01. PubMed PMID: 8626227.

416. Raymond J. The cyber file cabinet: dot-coms try to shift storage of medical records online. Am Demogr. 2000;22(7):38-40. Epub 2000/11/07. PubMed PMID: 11067697.

417. Richwine WB. No 'paradigm shift' needed at COMs. J Am Osteopath Assoc. 1993;93(11):1084, 202. Epub 1993/11/01. PubMed PMID: 8307790.

418. Sanders DG, Mooy CM. Ocular findings in cerebro-ocular-myopathy syndrome (COMS). A possible role of growth factors? Int Ophthalmol. 1993;17(4):223-8. Epub 1993/08/01. PubMed PMID: 8112970.

419. Schachat AP. Management of uveal melanoma: a continuing dilemma. Collaborative Ocular Melanoma Study Group. Cancer. 1994;74(11):3073-5. Epub 1994/12/01. PubMed PMID: 7954270.

420. Schreiber R. Department of medical illustration at COMS. J Osteopath (Kirksvill). 1963;70:48-50. Epub 1963/03/01. PubMed PMID: 13987021.

421. Sieving PA. Fifteen years of work: the COMS outcomes for medium-sized choroidal melanoma. Arch Ophthalmol. 2001;119(7):1067-8. Epub 2001/07/27. PubMed PMID: 11448330.

422. Singh AD, Kivela T. The collaborative ocular melanoma study. Ophthalmol Clin North Am. 2005;18(1):129-42, ix. Epub 2005/03/15. doi: 10.1016/j.ohc.2004.11.004. PubMed PMID: 15763198.

423. Straatsma BR. The Jules Francois Memorial lecture. The collaborative ocular melanoma study and management of choroidal melanoma. Bull Soc Belge Ophtalmol. 2002;(283):5-11. Epub 2002/06/13. PubMed PMID: 12058487.

424. Thomson RM, Furutani KM, Pulido JS, Stafford SL, Rogers DW. Modified COMS plaques for 125I and 103Pd iris melanoma brachytherapy. Int J Radiat Oncol Biol Phys. 2010;78(4):1261-9. Epub 2010/05/18. doi: 10.1016/j.ijrobp.2009.12.002. PubMed PMID: 20472360.

425. Wells CG, Bradford RH, Fish GE, Straatsma BR, Hawkins BS. Choroidal melanomas in American Indians. COMS Group. Collaborative Ocular Melanoma Study. Arch Ophthalmol. 1996;114(8):1017-8. Epub 1996/08/01. PubMed PMID: 8694711.

426. Zhang H, Martin D, Chiu-Tsao ST, Meigooni A, Thomadsen BR. A comprehensive dosimetric comparison between (131)Cs and (125)I brachytherapy sources for COMS eye plaque implant. Brachytherapy. 2010;9(4):362-72. Epub 2010/02/02. doi: 10.1016/j.brachy.2009.07.007. PubMed PMID: 20116342.

427. Caujolle J-P, Paoli V, Chamorey E, Maschi C, Baillif S, Herault J, et al. Local recurrence after uveal melanoma proton beam therapy: recurrence types and prognostic consequences. International Journal of Radiation Oncology, Biology, Physics. 2013;85(5):1218-24. doi: http://dx.doi.org/10.1016/j.ijrobp.2012.10.005. PubMed PMID: 23177670.

428. Dendale R, Lumbroso-Le Rouic L, Noel G, Feuvret L, Levy C, Delacroix S, et al. Proton beam radiotherapy for uveal melanoma: results of Curie Institut-Orsay proton therapy center (ICPO). International Journal of Radiation Oncology, Biology, Physics. 2006;65(3):780-7. PubMed PMID: 16647221.

429. Desjardins L, Lumbroso L, Levy C, Mazal A, Delacroix S, Rosenwald JC, et al. [Treatment of uveal melanoma with iodine 125 plaques or proton beam therapy: indications and comparison of local recurrence rates]. J Fr Ophtalmol. 2003;26(3):269-76. PubMed PMID: 12746603.

430. Gragoudas ES, Lane AM, Munzenrider J, Egan KM, Li W. Long-term risk of local failure after proton therapy for choroidal/ciliary body melanoma. Transactions of the American Ophthalmological Society. 2002;100:43-8; discussion 8-9. PubMed PMID: 12545676; PubMed Central PMCID: PMCPMC1358945.

431. Jensen AW, Petersen IA, Kline RW, Stafford SL, Schomberg PJ, Robertson DM. Radiation complications and tumor control after 125I plaque brachytherapy for ocular melanoma. International Journal of Radiation Oncology, Biology, Physics. 2005;63(1):101-8. PubMed PMID: 16111577.

432. Lane AM, Kim IK, Gragoudas ES. Proton irradiation for peripapillary and parapapillary melanomas. Archives of Ophthalmology. 2011;129(9):1127-30. doi: http://dx.doi.org/10.1001/archophthalmol.2011.228. PubMed PMID: 21911661.

433. Puusaari I, Heikkonen J, Summanen P, Tarkkanen A, Kivela T. Iodine brachytherapy as an alternative to enucleation for large uveal melanomas. Ophthalmology. 2003;110(11):2223-34. PubMed PMID: 14597534.
