# Supplementary material for: Cost-Effectiveness of Proton Beam Therapy for Intraocular Melanoma
Source: PLoS One. 2015 May 18;10(5):e0127814. doi: 10.1371/journal.pone.0127814 (PMC4436277; doi:10.1371/journal.pone.0127814)
Supplement: S1 Table — (DOCX) [file pone.0127814.s002.docx]

**S2 Table.** CPT-4 Codes Used for Medicare Reimbursement Rates for Cost Parameters

| **Description** | **CPT-4 Code** | **Quantity Billed†** | **Treatment Type** |
| --- | --- | --- | --- |
| Proton treatment delivery, intermediate | 77523 | 5 | Proton beam therapy |
| Computed tomography guidance for placement of radiation therapy fields | 77014 | 1 | Proton beam therapy |
| Therapeutic radiology simulation-aided field setting; complex | 77290 | 1 | Proton beam therapy, plaque brachytherapy |
| Therapeutic radiology treatment planning; complex | 77263 | 1 | Proton beam therapy, plaque brachytherapy |
| Radiation therapy dose plan (per field) | 77300 | 1 | Proton beam therapy, plaque brachytherapy |
| Intensity modulated radiotherapy plan | 77301 | 1 | Proton beam therapy |
| Treatment device; complex | 77334 | 1 | Proton beam therapy, plaque brachytherapy |
| Radiation physics consult | 77336 | 1 | Proton beam therapy, plaque brachytherapy |
| Therapeutic radiology port film(s) | 77417 | 1 | Proton beam therapy |
| Stereoscopic X-ray guidance (daily) | 77421 | 5 | Proton beam therapy |
| Radiation treatment management | 77427 | 1 | Proton beam therapy |

**S2 Table** (continued)

| **Description** | **CPT-4 Code** | **Quantity Billed†** | **Treatment Type** |
| --- | --- | --- | --- |
| Special treatment procedure | 77470 | 1 | Proton beam therapy, plaque brachytherapy |
| Special teletherapy port plan | 77321 | 1 | Proton beam therapy |
| Retrobulbar injection | 67500 | 10 | Proton beam therapy |
| Dexamethasone injection | J1100 | 10 | Proton beam therapy |
| Bupivacaine/Lido/Epi injection (unclassified drug) | J3490 | 10 | Proton beam therapy |
| Supervision, handling, loading of radiation source | 77790 | 1 | Plaque brachytherapy |
| Brachytherapy isodose plan; complex | 77328 | 1 | Plaque brachytherapy |
| Brachytherapy nonstrand I-125 >1.01 mCi | C2634 | 1 | Plaque brachytherapy |

Abbreviations: CPT, Current Procedure Terminology.

† Quantity Billed is the number of times each CPT code is charged to a single patient during their complete course of plaque brachytherapy or proton beam therapy
